# Bifunctional 3-Hydroxy-4-Pyridinones as Potential Selective Iron(III) Chelators: Solution Studies and Comparison with Other Metals of Biological and Environmental Relevance

**DOI:** 10.3390/molecules26237280

**Published:** 2021-11-30

**Authors:** Anna Irto, Paola Cardiano, Karam Chand, Rosalia Maria Cigala, Francesco Crea, Concetta De Stefano, Maria Amélia Santos

**Affiliations:** 1 Dipartimento di Scienze Chimiche, Biologiche, Farmaceutiche e Ambientali, Università di Messina, Viale F. Stagno d’Alcontres 31, 98166 Messina, Italy; pcardiano@unime.it (P.C.); rmcigala@unime.it (R.M.C.); fcrea@unime.it (F.C.); cdestefano@unime.it (C.D.S.); 2 Centro de Química Estrutural, Instituto Superior Técnico, Universidade de Lisboa, Av. Rovísco Pais 1, 1049-001 Lisbon, Portugal; kc4chemistry@gmail.com

**Keywords:** iron-chelation, chelating agents, 3-hydroxy-4-pyridinone, Fe-complexation, Cu-interaction, sequestering ability

## Abstract

The binding ability of five bifunctional 3-hydroxy-4-pyridinones towards Cu^2+^ and Fe^3+^ was studied by means of potentiometric and UV–Vis spectrophotometric measurements carried out at *I* = 0.15 mol L^−1^ in NaCl_(aq),_ *T* = 298.15 K and 310.15 K. The data treatments allowed us to determine speciation schemes featured by metal-ligand species with different stoichiometry and stability, owing to the various functional groups present in the 3-hydroxy-4-pyridinones structures, which could potentially participate in the metal complexation, and in the Cu^2+^ and Fe^3+^ behaviour in aqueous solution. Furthermore, the sequestering ability and metal chelating affinity of the ligands were investigated by the determination of p*L*_0.5_ and pM parameters at different pH conditions. Finally, a comparison between the Cu^2+^ and Fe^3+^/3-hydroxy-4-pyridinones data herein presented with those already reported in the literature on the interaction of Zn^2+^ and Al^3+^ with the same ligands showed that, from the thermodynamic point of view, the 3-hydroxy-4-pyridinones are particularly selective towards Fe^3+^ and could therefore be considered promising iron-chelating agents, also avoiding the possibility of competition, and eventually the depletion, of essential metal cations of biological and environmental relevance, such as Cu^2+^ and Zn^2+^.

## 1. Introduction

Copper (Cu) and iron (Fe) are essential metals for plants, animals and humans, ensuring their normal biochemical and physiological functions [1,2]. In healthy situations, living organisms are provided with homeostatic mechanisms and buffers to keep normal metal concentration levels and to avoid anomalous phenomena, as metal decompartmentalization, release and mobilization [3].

In plants, Cu plays important roles in photosynthetic and respiratory electron transport processes, occurring in chloroplasts and mitochondria. It participates in the oxidative stress protection, acts as cofactor of many enzymes and plays key functions in the cell wall metabolism, namely for Fe-mobilization, oxidative phosphorylation and the biogenesis of the molybdenum cofactor [4]. In the human body, copper favours the normal development of the brain and nervous system and maintains a fair level of white blood cells. Cu is also necessary to keep the muscle tone and functions; it is involved in the formation of red blood cells and in the processes of absorption and transport of iron (Fe^3+^) in the body. Furthermore, the generation of cellular energy in the form of ATP into the mitochondria depends on the participation of a copper-containing enzyme.

As regards iron, in living organisms it plays essential functions in metabolic processes, like photosynthesis, respiration and DNA synthesis [5]. In plants, mainly present in ferric (Fe^3+^) form [6], it participates in the chlorophyll production [7], being also necessary for nitrogen fixation processes and for plants growth [8]. It is detected in iron-containing heme-proteins, like cytochromes present in electron transfer systems within the mitochondria and chloroplasts, and also in non-heme proteins, as ferredoxin. Iron can become a toxic element for soils and plants when it is accumulated at significant concentration levels. In these matrices, Fenton reaction (iron redox cycle) can produce reactive oxygen species (ROS) like -OH radicals [3], which are able to damage DNA, lipids and proteins so that effects like soils bronzing and leaves stippling may occur. As an example, the reason for colour alterations in leaves could be the plants’ synthesis of enzymes aimed to control the free radicals effects, like in the case of basil, tomato, impatiens and phlox plants [8,9]. In addition, Fe^3+^ could also compete with Cu^2+^ and Zn^2+^ in its uptake and transport within plant cells [10]. In humans and other mammals, iron is a fundamental constituent of myoglobin and haemoglobin, proteins able to transport oxygen along the body. It is also important for the normal functions of hormones and cells, also being the cofactor of many enzymes, like in cytochromes B5, C and P450 [8]. Similarly to what was observed in plants and soils, in humans and mammals iron excess can also be very toxic. In fact, a Fenton reaction produces hydroxyl radicals which can react with nucleic acids, proteins, sugars and lipids, leading to pathological situations such as DNA and RNA damage, proteins and sugars oxidation and lipid peroxidation, respectively. Unfortunately, since physiological mechanisms for iron elimination do not exist, acute and chronic effects due to its overload can be often observed [3]. Examples of harmful effects provoked by iron accumulation are the deterioration of the gastric and intestinal mucosae, Bantu siderosis, cardiovascular diseases, neurodegenerative disorders and carcinogenic risks [8]. Iron overload could also cause haemoglobinopathy diseases such as transfusional hemosiderosis, owing to the metal parenteral administration for the treatment of *β*-thalassemia major, or hemochromatosis, a genetic disorder correlated with iron over-absorption [3].

In this light, many research efforts have been devoted to the development of new strategies aimed to remove metals from different matrices without side effects. All the green and sustainable approaches developed for metals extraction from environmental matrices are called “*chelation technologies*” [11], whereas the treatment of human diseases related to the metal intoxication is called “*chelation therapy*”. This last approach is based on the administration of chelating agents to patients suffering from metals overload, inducing their sequestration and systemic excretion [12,13]. As regards iron chelation, potential chelating agents should fulfil some criteria, such as: absence of toxicity of the chelators and the corresponding iron-complexes, economic availability, drug-likeness properties, selectivity towards the metal cation of interest without involving the depletion of essential components, good intestinal absorption, oral activity, affinity towards biological membranes, good bioavailability to the target cells, higher metal chelating capacity and specificity with respect to the commercial molecules [14].

The bidentate chelators 3-hydroxy-4-pyridinones (3,4-HPs) match these criteria and therefore this family of compounds have been considered as promising drug candidates. They are characterized by an aromatoid *N*-heterocyclic ring, containing an exocyclic pair of electron donor atoms (*O*-*O*), featured by a ketone and a hydroxyl substituent groups in the *ortho* position, which confers them a high affinity towards divalent and trivalent metal cations [15]. In fact, a 3,4-HP derivative, namely the 1,2-dimethyl-3-hydroxy-4-pyridinone, (Deferiprone, *DFP*) is approved as an orally active chelating drug for the treatment of iron overload patients. Since its disclosure and later approval [16,17], many 3,4-HP derivatives, have been developed with the aim of overcoming some *DFP* drawbacks and efficacy improving [3]. Following this strategy, we have recently explored a small family of compounds, namely bifunctional bidentate 3,4-HP ligands, with the aim of improving their lipophilic–hydrophilic balance, bioavailability and affinity towards biological membranes, as well as their chelating efficacy towards Fe^3+^ with respect to the commercially available chelating agents [13,18].

**Figure 1:**
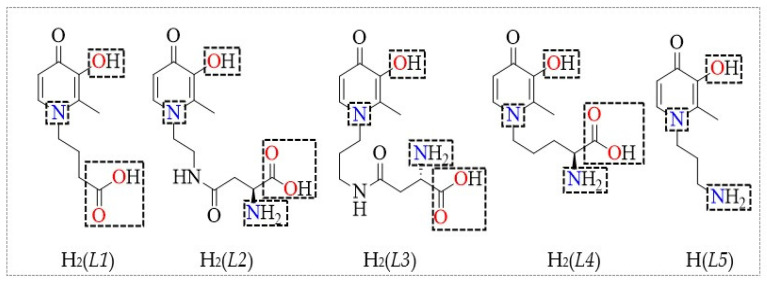
Structures of the 3-hydroxy-4-pyridinone ligands, with the protonable groups enclosed in dotted rectangles.

Herein, pursuing our previous strategy, we present the results of a potentiometric and UV–Vis (Ultraviolet–Visible) spectrophotometric investigation on the interaction of five bifunctional 3-hydroxy-4-pyridinones (Figure 1) with Cu^2+^ and Fe^3+^, metal cations with a *borderline* and a *hard* character, respectively, that are carried out at *I* = 0.15 mol L^−1^ in NaCl_(aq)_, *T* = 298.15 K and 310.15 K. Furthermore, the obtained thermodynamic data are compared with those already reported in the literature on the binding ability and chelating affinity of the five ligands towards Zn^2+^ [19] and Al^3+^ [13], also featured by *borderline* and *hard* behaviour, respectively, at the same experimental conditions. The aim of this work was to evaluate whether the 3-hydroxy-4-pyridinones under study could be exploited as selective chelating agents for the treatment of Fe^3+^ overload in humans or, alternatively, in environmental matrices. Another relevant issue worth investigating was to ascertain whether, from a thermodynamic point of view, along with an effective Fe^3+^-sequestration, a significant competition, and possibly depletion, of divalent metals of biological and environmental relevance such as Cu^2+^ and Zn^2+^ may occur, despite the different charge density, acid–base behaviour [20,21,22] and ionic radius [23].

Along the text, in the tables and figures, the five 3-hydroxy-4-pyridinones under study will be indicated with the abbreviations:

H_2_(*L1*) = 4-(3-hydroxy-2-methyl-4-oxopyridin-1(4*H*)-yl)butanoic acid;

H_2_(*L2*) = (*S*)-2-amino-4-((2-(3-hydroxy-2-methyl-4-oxopyridin-1(4*H*)-yl)ethyl)amino)-4-oxobutanoic acid;

H_2_(*L3*) = (*S*)-2-amino-4-((3-(3-hydroxy-2-methyl-4-oxopyridin-1(4*H*)-yl)propyl)amino)-4-oxobutanoic acid;

H_2_(*L4*) = (*S*)-2-amino-5-(3-hydroxy-2-methyl-4-oxopyridin-1(4*H*)-yl)pentanoic acid;

H(*L5*) = 1-(3-aminopropyl)-3-hydroxy-2-methylpyridin-4(1*H*)-one.

## 2. Results and Discussion

### 2.1. Equilibria for the Formation of Metal-Ligand Species

The formation or stability constants of the metal-ligand species are expressed considering the following stepwise (1) and overall (2) equilibria:

pM^n+^ + H_r_*L*_q_^-(zq-r)^ = M_p_H_r_*L*_q_^(pn+r-qz)^    *K*_pqr_ (1)

pM^n+^ + q*L*^z-^ + rH^+^ = M_p_H_r_*L*_q_^(pn+r-qz)^    *β*_pqr_ (2)

The equilibrium constants, concentrations and ionic strengths are expressed in the molar (*c*, mol L^−1^) concentration scale.

### 2.2. Synthesis of the Ligands

The five 3-hydroxy-4-pyridinones (Figure 1) have been synthesized and characterized in the neutral form (H_r_*L*^0^), following procedures already reported in the literature [13].

### 2.3. Acid–Base Properties of Ligands and the Metal Cations

The 3-hydroxy-4-pyridinones under study are featured by different protonable groups highlighted in Figure 1 with dotted rectangles. The ligands’ structure consists of a hydroxyl group as substituent on the *N*-heterocyclic ring, a -NH_2_ and/or -COOH on the alkyl chain and a pyridinone nitrogen atom (proton provided by an excess of inorganic acid) [13], each of them with different acidity. The 3-hydroxy-4-pyridinones protonation constants have been already reported in the literature at *I* = 0.15 mol L^−1^ in NaCl_(aq)_, *T* = 298.15 and 310.15 K (Table S1) [13].

The hydrolytic constants of Cu^2+^ and Fe^3+^ have already been published [20,21,22]. In the case of Fe^3+^, the solubility product related to the formation of Fe(OH)_3_^0^_(s)_ sparingly soluble species has been also considered [21].

### 2.4. Metal-Ligand Studies

The elaboration of potentiometric and UV–Vis spectrophotometric data on the binding ability of the ligands towards Cu^2+^ and Fe^3+^ allowed us to determine various speciation schemes, based on the different acid–base properties of the 3,4-HPs in NaCl_(aq)_, the hydrolytic behaviour and the charge density of the metal cations. The best possible speciation models were selected on the basis of criteria such as the simplicity and probability of the model, the species formation percentages in the whole investigated pH, the statistical parameters (like the standard deviation on equilibrium constants and on the fitting values), the corresponding ratios between single variances compared with those from the accepted model. The high number of experiments carried out and experimental points collected allowed for the consideration of differences in variance between the accepted model and others to be significant.

In the case of the Cu^2+^ and Fe^3+^/(3,4-HPs) interactions investigated with both of the mentioned analytical techniques, an average of the potentiometric and UV–Vis stability constants was calculated with the aim of describing the systems in a more complete way, considering a wide range of metal and ligand concentrations used, namely *c* ~ 10^−3^ mol L^−1^ and ~10^−4–^10^−5^ mol L^−1^, for potentiometric and UV–Vis spectrophotometric measurements, respectively.

#### 2.4.1. Cu^2+^/(3,4-HPs) Systems

For each investigated Cu^2+^/(3,4-HPs) system, the treatment of potentiometric and UV–Vis spectrophotometric data recorded at *I* = 0.15 mol L^−1^ in NaCl_(aq)_, *T* = 298.15 K and pH ranges 2.0–10.0 and 2.0–11.0, respectively, allowed us to obtain speciation models featured by complex species with 1:1 stoichiometry (Cu*L*^(2-z)^) and different protonation degrees (CuH_2_*L* ^(4-z)^, CuH*L*^(3-z)^). The experimental formation constants obtained by each analytical techniques are in accordance with each other, and they are reported in Table 1. As can be observed in Table 1, at the mentioned experimental conditions a trend of the complexes’ stability can be observed, based on the common species Cu*L*^(2-z)^: Cu(*L3*)^0^_(aq)_ > Cu(*L4*)^0^_(aq)_ > Cu(*L2*)^0^_(aq)_ > Cu(*L5*)^+^ > Cu(*L1*)^0^_(aq)_. This trend could be explained considering that the stability of the Cu^2+^/(3,4-HPs) species may be favoured by the concomitant presence of the extra-functional groups in the 3,4-HP ligand molecules, namely -COOH, -NH_2_ and -CHNH_2_COOH bearing groups in the alkyl chain bound to the *N*-heterocyclic ring, which, in some cases have also inserted an amide moiety (H_2_(*L2*), H_2_(*L3*)) (Figure 1). Generally, the complexes with higher stability are those where the 3,4-HP ligands are extra-functionalized with α-amino-carboxylic groups (H_2_(*L2*), H_2_(*L3*), H_2_(*L4*)), probably due to their inherent chelating capacity [24]. The different length of the alkyl moiety is also another factor influencing the stability of the species; in fact, from the comparison between the data obtained for Cu(*L2*)^0^_(aq)_ and Cu(*L3*)^0^_(aq)_ species, which only differ in the ligand structures by an additional -CH_2_ group present in the H_2_(*L3*) alkyl chain (Figure 1), a decrease of the formation constants with alkyl moiety length decreasing can be observed (Table 1). Furthermore, from the comparison among the Cu(*L1*)^0^_(aq)_ and Cu(*L5*)^+^ stability constants it can be observed that the ligand featured by the only amino group (H(*L5*)) in the alkyl chain forms Cu^2+^ complexes with higher stability than the carboxylic-3-hydroxy-4-pyridinone (H_2_(*L1*)), a trend which is in accordance with data reported in the literature [24,25,26] on the interactions of alkylamines and carboxylic acids towards Cu^2+^, also following the Pearson’s principle of “*hard* and *soft* acids and bases” theory (HSAB) for ligand-metal preferences [27,28,29].

**Figure 2:**
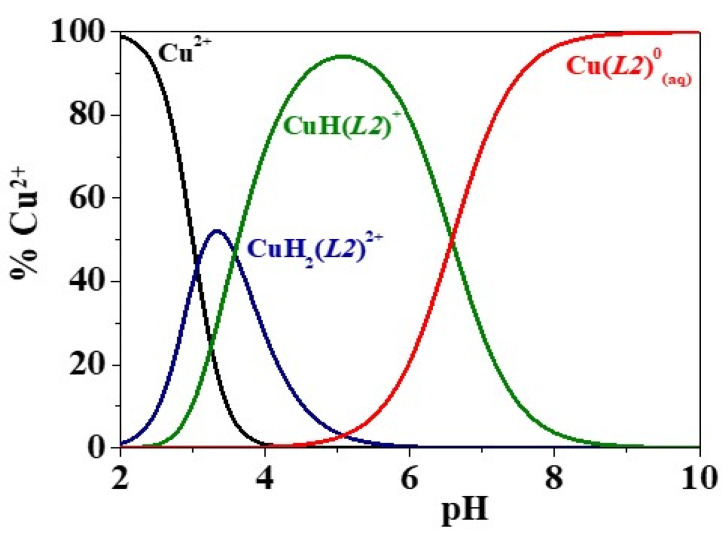
Distribution diagram of the Cu^2+^/H_2_(*L2*) system at *T* = 298.15 K, *I* = 0.15 mol L^−1^ in NaCl_(aq)_, *c*_Cu_^2+^ = 5.0·10^−4^ mol L^−1^ and *c*_ligand_ = 1.5·10^−3^ mol L^−^^1^.

A further comparison between the speciation of the different Cu^2+^/3-hydroxy-4-pyridinone systems may be performed based on the distribution diagrams reported in Figure 2, for H_2_(*L3*)*,* and Figure S1, for the other ligands. In the case of H_2_(*L1*) (Figure S1a), the diagram shows that the formation of the CuH(*L1*)^+^ and Cu(*L1*)^0^_(aq)_ species reaches the 68% and 99% maximum percentages at pH ~ 3.9 and pH ~ 6.6, respectively. As regards the distribution of Cu^2+^/H_2_(*L2*) (Figure 2), H_2_(*L3*) (Figure S1b) and H_2_(*L4*) (Figure S1c) species, the metal-ligands complexation occurs up to pH ~ 3.2–3.3 with the formation of CuH_2_*L*^2+^ species exceeding the 52% formation. The CuH*L*^+^ complex achieves more than the 86% formation at pH ~ 5.0–5.1 for H_2_(*L2*) and H_2_(*L4*), pH ~ 5.5 for H_2_(*L3*). The 1:1 stoichiometry complex starts to form at pH ~ 4.0, 4.5 and 3.6 and reaches more than the 99% formation at pH ~ 8.8, 9.1 and 8.2, for H_2_(*L2*)_,_ H_2_(*L3*) and H_2_(*L4*), respectively. In the case of the Cu^2+^/H(*L5*) system (Figure S1d), the formation of the Cu(*L5*)H^2+^ and Cu(*L5*)^+^ species reaches their maximum percentages at pH ~ 5.4 and pH ~ 9.6, respectively.

**Figure 3:**
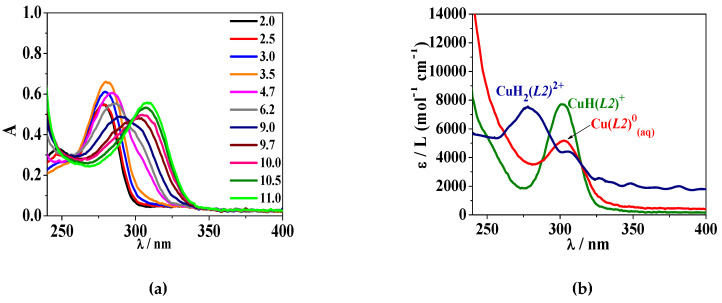
(**a**) UV–Vis absorption profile of the Cu^2+^/H_2_(*L2*) system at different pH values and (**b**) calculated molar absorptivity of CuH_2_(*L2)*^2+^, CuH(*L2)*^+^, Cu(*L2)*^0^_(aq)_ species at *T* = 298.15 K, *I* = 0.15 mol L^−1^ in NaCl_(aq)_, *c*_Cu_^2+^ = 2.0·10^−5^ mol L^−1^ and *c*_ligand_= 8.5·10^−5^ mol L^−1^.

Concerning the UV–Vis spectrophotometric behaviour, a representative example of the spectra recorded for the Cu^2+^/H_2_(*L2*) system is shown in Figure 3a. An absorption band with λ_max_ = 278 nm can be observed at pH ~ 2.0–2.5. Its intensity increases with the pH, up to pH ~ 3.5, and then it starts to decrease, up to pH ~ 9.0–9.7, undergoing a bathochromic shift. Above pH ~ 10.0, the band intensity raises again with a subsequent red shift, up to pH ~ 11.0. The deconvolution of the UV–Vis data allowed us to calculate the molar absorptivity (ε/L (mol^−1^ cm^−1^)) values for each metal-ligand species. As an example, a graphical representation of the ε determined for the Cu^2+^/H_2_(*L2*) system is reported in Figure 3b. At *I* = 0.15 mol L^−1^ in NaCl_(aq)_, *T* = 298.15 K, the molar absorptivities are: ε_max_(CuH_2_(*L2*)^2+^) = 7514 at λ_max_ = 280 nm, ε_max_(CuH(*L2*)^+^) = 7813 at λ_max_ = 302 nm, ε_max_(Cu(*L2*)_(aq)_) = 5222 at λ_max_ = 304 nm.

#### 2.4.2. Fe^3+^/(3,4-HPs) Systems

The investigation on the binding ability of the H_2_(*L1*), H_2_(*L2*), H_2_(*L4*) and H(*L5*) ligands towards Fe^3+^ was carried out by potentiometric titrations at *I* = 0.15 mol L^−1^ in NaCl_(aq)_ and *T* = 298.15 K. In the case of H_2_(*L2*) and H(*L5*), UV–Vis experiments were performed also at the same ionic strength and *T* = 310.15 K. The data treatment allowed for the determination of FeH*L*^(4-z)^, Fe*L*^(3-z)^ and Fe*L*_2_^(3-2z)^ species in the pH range 2.0–5.0, due to the formation of a red colour precipitate, attributable to the sparingly soluble Fe(OH)_3_^0^_(s)_ species [21]. This limitation was overcome by spectrophotometric titrations performed at more diluted conditions, thus allowing to explore the measurements in a wider pH range (2.0–9.1).

The stability constants determined at the different experimental conditions are reported in Table 2. The values obtained by the two analytical techniques are in quite good agreement. Similarly to the Cu^2+^/(3,4-HPs) studies, in this case the data average was also calculated.

Based on the common Fe*L*^(3-z)^ species, the following trend can be observed: Fe(*L4*)^+^ > Fe(*L2*)^+^ > Fe(*L5*)^2+^ > Fe(*L1*)^+^ at *T* = 298.15 K. The stability of the Fe^3+^/(3,4-HP) species is favoured by the concomitant presence of the extra-functional groups on the 3-hydroxy-4-pyridinone derivatives, in particular the α-amino-carboxylic groups in alkyl chain ((H_2_(*L2*) and H_2_(*L4*), Figure 1) [24]. Furthermore, from the comparison among the Fe(*L1*)^+^ and Fe(*L5*)^+^ formation constant values, it can be observed that the ligand featured by the only amino group (H(*L5*)) in the alkyl chain forms Fe^3+^ complexes with higher stability than the carboxylic-3-hydroxy-4-pyridinone (H_2_(*L1*)), in accordance with literature data [24] reported on the interaction of alkylamines and carboxylic acids towards Fe^3+^.

In the case of the H_2_(*L2*) and H(*L5*) ligands, the formation constants were also determined at *I* = 0.15 mol L^−1^ in NaCl_(aq)_ and *T* = 310.15 K, as reported in Table 2: the obtained values increase with temperature.

**Figure 4:**
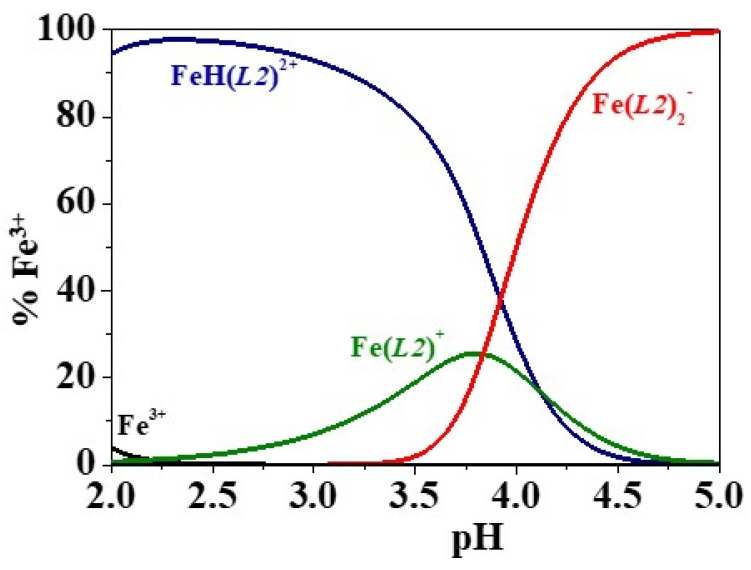
Distribution diagram of the Fe^3+^/H_2_(*L2*) system at *T* = 298.15 K, *I* = 0.15 mol L^−1^ in NaCl_(aq)_, *c*_Fe_^3+^ = 5.0·10^−4^ mol L^−1^ and *c*_ligand_ = 1.1·10^−3^ mol L^−1^.

A further deepening on the speciation of the different Fe^3+^/(3,4-HP) systems may be performed considering the distribution diagrams drawn from potentiometric data at *I* = 0.15 mol L^−1^ in NaCl_(aq)_ and *T* = 298.15 K, as reported in Figure 4 for H_2_(*L2*) and in Figure S2 for H_2_(*L1*), H_2_(*L4*) and H(*L5*).

In the case of H_2_(*L1*) (Figure S2a), the diagram shows the FeH(*L1*)^2+^ and Fe(*L1*)^+^ species reaching the maximum percentages of 93% and 19% at pH ~ 2.8 and 3.5, respectively. Introducing the solubility product of Fe(OH)^0^_3(s)_ [21] in the speciation model in the HySS programme [30], used to calculate the formation percentages and to represent the distribution diagrams, the formation of the sparingly soluble species should occur at pH ~ 3.5, hindering the possible formation of the Fe(*L1*)_2_^-^ species which should only start to form at the mentioned pH value. However, since the precipitation was experimentally observed at pH ~ 5.0, the formation of the Fe(*L1*)_2_^-^ species in the pH range 3.5–5.0 could be considered as a probable complex, and therefore it was reported in Table 2. Regarding the species distribution of Fe^3+^/H_2_(*L2*) (Figure 4), Fe^3+^/H_2_(*L4*) (Figure S2b) and Fe^3+^/H(*L5*) (Figure S2c) species, the FeH*L*^(4-z)^ species achieves 99% formation at pH ~ 2.3–2.4. The 1:1 stoichiometry complex reaches 26%, 37% and 7% formation at pH ~ 3.8, 3.7, 3.2 for H_2_(*L2*)_,_ H_2_(*L4*) and H(*L5*)_,_ respectively. As regards the Fe*L*_2_^(3-2z)^ complex, it starts to form from pH ~ 3.2 with ligands as H_2_(*L2*) and H_2_(*L4*), from pH ~ 2.6 with H(*L5*).

**Figure 5:**
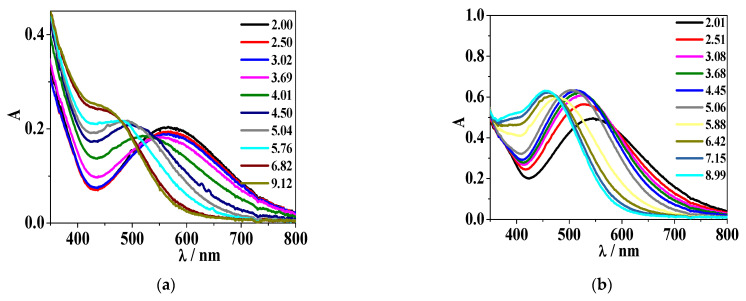
UV–Vis absorption profiles of the Fe^3+^/H_2_(*L2*) system at *I* = 0.15 mol L^−1^ in NaCl_(aq)_, *T* = 298.15 K and at different pH values. (**a**) *c*_Fe3+_ = 2.5·10^−4^ mol L^−1^, *c*_ligand_ = 1.8·10^−4^ mol L^−1^; (**b**) *c*_Fe3+_ = 2.5·10^−4^ mol L^−1^, *c*_ligand_ = 5.8·10^−4^ mol L^−1^.

**Figure 6:**
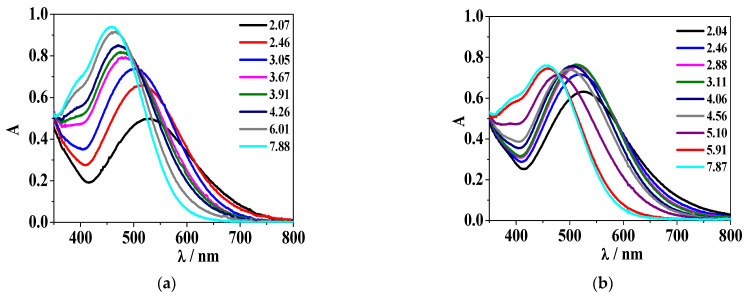
UV–Vis absorption profiles of the Fe^3+^/H*(L5)* system at *I* = 0.15 mol L^−1^ in NaCl_(aq)_, different temperatures and pH values. (**a**) *T* = 298.15 K, *c*_Fe3+_ = 2.0·10^−4^ mol L^−1^, *c*_ligand_ = 6.4·10^−4^ mol L^−1^; (**b**) *T* = 310.15 K, *c*_Fe3+_ = 2.5·10^−4^ mol L^−1^, *c*_ligand_ = 5.7·10^−4^ mol L^−1^.

Figure 5, Figure 6, Figures S3 and S4 show the UV–Vis behaviour of Fe^3+^/H_2_(*L2*) and Fe^3+^/H(*L5*) systems at different component concentration and temperatures.

For the Fe^3+^/H_2_(*L2*) system (Figure 5 and Figure S3), a band with λ_max_ = 568 nm is observed at pH ~ 2.0, followed by an intensity decrease at pH ~ 3.7. A first band hypsochromic shift (λ_max_ = 510 nm) and an absorbance increase occurs at pH ~ 4.5. Then, a second blue shift and a band is observed with λ_max_ = 460 nm from pH ~ 6.1–7.1, depending on the experimental conditions, up to the formation of precipitate, which hindered further investigations.

In the case of the Fe^3+^/H(*L5*) system, at metal/ligand stoichiometric conditions (Figure S4), the mentioned band with λ_max_ = 568 nm at pH ~ 2.0, as well as its two hypsochromic shifted bands (λ_max_ = 510 nm, 460 nm) at pH ~ 4.9 and 5.9–6.0, respectively, are observed. For *c*_Fe3+_/*c*_ligand_ = 1/2 and *c*_Fe3+_/*c*_ligand_ = 1/3 (Figure 6), the first recorded band is featured by λ_max_ = 536 nm at pH ~ 2.0, with a blue shift occurring at pH ~ 4.9 with a band at λ_max_ = 515 nm, whilst the last blue shift corresponds to a band at λ_max_ = 460 nm, similarly to the previous described spectra.

**Figure 7:**
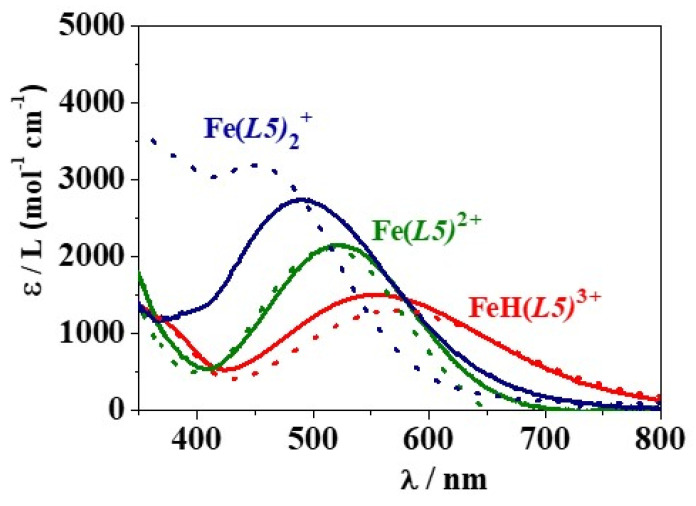
Graphical representation of molar absorptivity of Fe^3+^/H*(L5)* species at *I* = 0.15 mol L^−1^ in NaCl_(aq)_, *T* = 298.15 K (solid line) and 310.15 K (dot line).

The deconvolution of the UV–Vis spectrophotometric data allowed us to calculate the molar absorptivity (ε/L (mol^−1^ cm^−1^)) values for each metal-ligand species. Graphical representations of the ε determined for the Fe^3+^/H_2_(*L2*) and Fe^3+^/H(*L5*) systems are reported in Figure S5 and Figure 7, respectively, at *I* = 0.15 mol L^−1^ in NaCl_(aq)_ and different temperatures. As a representative example, the calculated molar absorptivities for the Fe^3+^/H(*L5*) species are: ε_max_(FeH(*L5)*_3+_) = 1440 at λ_max_ = 571 nm, ε_max_ (Fe(*L5)*^2+^) = 2160 at λ_max_ = 520 nm, ε_max_(Fe(*L5)* ^2+^) = 2736 at λ_max_ = 492 nm at *T* = 298.15 K; ε_max_(FeH(*L5)*_3+_) = 1293 at λ_max_ = 578 nm, ε_max_(Fe(*L5)* ^2+^) = 2124 at λ_max_ = 518 nm, ε_max_(Fe(*L5)*^2+^) = 3006 at λ_max_ = 460 nm at *T* = 310.15 K.

### 2.5. Literature Data Comparison

From the best of our knowledge, no studies have been reported on the Cu^2+^/(3,4-HP) systems. Two papers have been published by Santos et al. [31,32] on the binding ability of the same H_2_(*L1*) and H_2_(*L4*) ligands (Figure 1) towards Fe^3+^ at *I* = 0.10 mol L^−1^ in KNO_3(aq)_ and *T* = 298.15 K. The authors determined a speciation scheme featured by FeH_r_*L*_q_^(3+r-qz)^ (q, r = 1–3) species with different stoichiometry, including FeH*L*^2+^. This complex was also reported in the current work for the same two ligands (Table 2), and so a comparison between the experimental and literature data can be made. The stability constants determined by Santos et al. are log*K*_111_ = 9.58 for H_2_(*L1*) [32] and log*K*_111_ = 15.16 for H_2_(*L4*) [31] (Table S2). The value obtained for H_2_(*L4*) is in good accordance with the log*K*_111_ = 15.21 (Table 2) presented in this paper at *I* = 0.15 mol L^−1^ in NaCl_(aq)_ and *T* = 298.15 K. However, the value previously reported for H_2_(*L1*) is slightly higher than the value determined herein (log*K*_111_ = 7.42, Table 2).

Some other comparisons could also be made considering metal-ligand investigations on compounds with similar structures and functional groups (Figure S6) with respect to the 3,4-HP ligands under study. Nevertheless, some little differences in the ligand structures, discrepancies between the experimental conditions and, in particular, the different approaches sometimes used by the authors for the data treatment (determination of ligands’ acid–base properties, apparent neglect or very few information reported on the metals’ hydrolytic behaviour), make it difficult to establish a direct comparison among the stability constants. However, an attempt of comparison could be performed, considering the log*K*_110_ values reported in this paper for the M*L*^(n-z)^ species and the data published in the literature for complexes with the same stoichiometry.

Concerning the Cu^2+^/ligand systems, the stability constants reported in Table 1, for Cu(*L1*)^0^_(aq)_, Cu(*L2*)^0^_(aq)_, Cu(*L3*)^0^_(aq)_, Cu(*L4*)^0^_(aq)_ and Cu(*L5*)^+^ species at *I* = 0.15 mol L^−1^ in NaCl_(aq)_ and *T* = 298.15 K, can be compared with those published for the Cu*L*^(2-z)^ complexes determined with Deferiprone (*DFP*) [33,34] and *AcNPrHP* ([35] (Figure S6, Table S2) at *I* = 0.10 mol L^−1^ in KCl_(aq)_ and the same temperature. As regards the Cu(*L1*)^0^_(aq)_ species, its stability is three orders of magnitude lower than the mentioned literature data. In the case of the other four 3-hydroxy-4-pyridinones, the copper-experimental values were found to be about two (Cu(*L5*)^+^) and five (Cu(*L2*)^0^_(aq)_, Cu(*L3*)^0^_(aq)_, Cu(*L4*)^0^_(aq)_) logarithmic units higher (Table 1), respectively, with respect to the literature ones (Table S2) [33,34,35]. The formation constant published for the Cu^2+^/*L*-Aspartic acid (*Asp*) 1:1 stoichiometry species (Table S2) [22] can be compared with the corresponding H_2_(*L2*) and H_2_(*L3*) derivatives. In this case, the experimental values (Table 1) are six orders of magnitude higher with respect to the literature data [22].

As regards the Fe^3+^/(3,4-HPs) systems, the stability of the Fe(*L1*)^+^, Fe(*L2*)^+^, Fe(*L4*)^+^ and Fe(*L5*)^2+^ species (Table 2), at *I* = 0.15 mol L^−1^ in NaCl_(aq)_ and *T* = 298.15 K and 310.15 K, can be compared with the literature data (Table S2) reported for the Fe(*DFP*)^2+^ complex at *I* = 0.10 mol L^−1^ in KCl_(aq)_, at the same temperatures [33,34,36]. The Fe(*L1*)^+^ experimental value is about one–two logarithmic units lower with respect to the mentioned literature data. The Fe(*DFP*)^2+^ stability constants [33,34,36] are instead about six–seven orders of magnitude lower than the Fe(*L2*)^+^, Fe(*L4*)^+^ and Fe(*L5*)^2+^ stability constants reported in Table 2. Similar observations can be made for the formation constants published for the Fe(*Asp*)^2+^ species, at *I* = 1.00 mol L^−1^ in a Na^+^ background electrolyte, *T* = 293.15 K [22], as well as for Fe(*Orn*)^+^ (*L*-Ornithine) at *I* = 0.10 mol L^−1^ in NaClO_4(aq)_, at the same temperature [37], which can be compared with their H_2_(*L2*) and H_2_(*L4*) derivatives, respectively. In particular, in the case of Fe(*L2*)^+^ species (Table 2), the metal-ligand stability is about eleven logarithmic units higher with respect to the Fe(*Asp*)^2+^ one [22]. The Fe(*L4*)^+^ experimental value (Table 2) is almost fourteen orders of magnitude higher than the Fe(*Orn*)^+^ literature constant [37]. The data reported in the literature (Table S2) for other 3,4 HP analogues (H_2_*Si*, i = 1–3), for the Fe(*S1*)^2+^ complex at *I* = 0.10 mol L^−1^ in KNO_3(aq)_ [38] and for the Fe(*S2*)^+^ and Fe(*S3*)^+^ ones at the same ionic strength and temperature but in a MOPS (3-(*N*-morpholino)propanesulphonic acid) buffer at pH = 7.4 [39], present a stability six–height orders of magnitude lower than those observed for all the Cu^2+^/(3,4-HPs) 1:1 stoichiometry species, with the exception of the Fe(*L1*)^+^ experimental value, which was between five and seven logarithmic units higher with respect to the literature data [38,39].

Overall, the generally much higher values found for the stability of the 1:1 metal complex with the ligands bearing a terminal α-amino-carboxylic group (H_2_(*L2*), H_2_(*L3*), H_2_(*L4*)) may be mainly attributed to the probable co-adjuvation of the main hydroxypyridinone (*O*,*O*) metal coordination by the (*N*,*O*) glycine type coordination, and also the inserted amide bond, which can further interfere in the length and rigidity of the linker between both main groups.

### 2.6. Sequestering Ability

The evaluation of the sequestering ability of the 3-hydroxy-4-pyridinones towards Cu^2+^ and Fe^3+^ can be performed by calculating the p*L*_0.5_ empirical parameter which represents the total ligand concentration required for the 50% sequestration of a metal cation if present in trace amount in solution. The p*L*_0.5_ can be described using a sigmoidal type Boltzmann equation, with asymptotes equal to 1 for p*L*→−∞ and 0 for p*L* → +∞ (Equation (3)):

(3) $$x_{M}=\frac{1}{{1+10}^{{(pL-pL}_{0.5})}}$$

where *x*_M_ is the mole fraction of metal cation complexed by the ligand, p*L* = −log *c*_L_ and p*L*_0.5_ = −log *c_L_*, if *x*_M_ = 0.5. The evaluation of the sequestering ability is very important for detoxification, remediation of polluted systems and water treatment processes, requiring the use of a chelating agent with the aim of trying to optimize the working conditions. A more detailed description of the p*L*_0.5_ determination, its importance and other possible applications is reported in the literature [40].

The study of the sequestering ability of the ligands towards Cu^2+^ and Fe^3+^ was performed at *I* = 0.15 mol L^−1^ in NaCl_(aq)_, *T* = 298.15 K and different pHs. In the case of Fe^3+^/H_2_(*L2*) and Fe^3+^/H(*L5*) systems, the p*L*_0.5_ was also determined at the same ionic strength and *T* = 310.15 K.

From the analysis of the data reported in Table 3 and Figure S7a for the Cu^2+^/*L2* system, it can be concluded that the sequestering ability increases with pH, probably due to the gradual ligand deprotonation, which favours the metal-ligand electrostatic interaction. At pH = 7.4 (physiological value), *I* = 0.15 mol L^−1^ in NaCl_(aq)_ and *T* = 298.15 K, the p*L*_0.5_ trend is: H_2_(*L2*) ≥ H_2_(*L3*) > H_2_(*L4*) > H(*L5*) > H_2_(*L1*) (Table 3, Figure S7b). As already observed for the stability constants, the sequestering ability is also influenced by the presence in the ligands structure of the -CO_2_H, -NH_2_, -CHNH_2_CO_2_H [24] and, possibly, the amidic moiety in the alkyl chain. In addition, the p*L*_0.5_ value obtained for the amino-3-hydroxy-4-pyridinone (H(*L5*)) is slightly higher than the one calculated for H_2_(*L1*) (terminal -CO_2_H group), highlighting a better Cu^2+^ sequestration by the ligand featured by the terminal -NH_2_ group (H(*L5*)) with respect to the carboxylic one [24,25,26].

**Figure 8:**
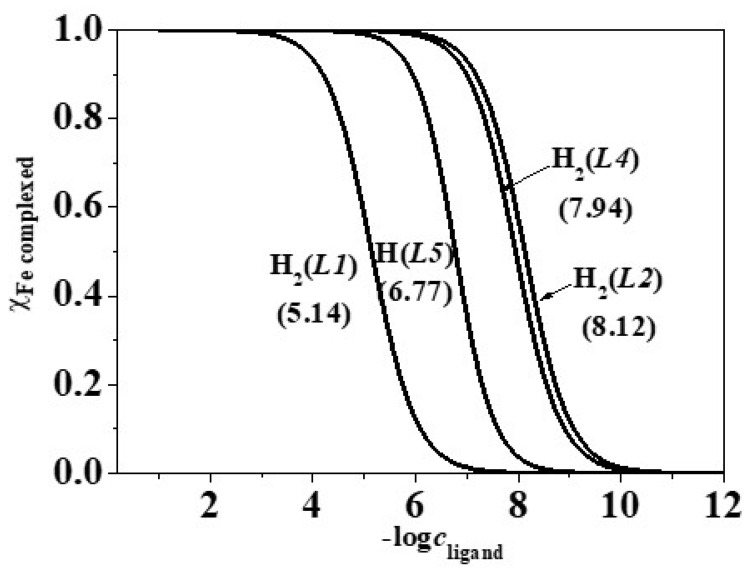
Sequestration diagram of the ligands towards Fe^3+^ at pH = 4.0, *I* = 0.15 mol L^−1^ in NaCl_(aq)_ and *T* = 298.15 K.

As regards the iron-containing systems, the formation of precipitate at pH ~ 5.0 during the potentiometric measurements allowed us to evaluate the sequestering ability of the ligands in a quite narrow pH range. As can be observed in Figure 8, the p*L*_0.5_ trend at pH = 4.0 is: H_2_(*L2*) (8.12) > H_2_(*L4*) (7.94) > H(*L5*) (6.77) > H_2_(*L1*) (5.14), confirming that, analogously to what was observed for the stability constants, the sequestration is mainly favoured by the presence in the ligand structures of the amide-amino-carboxylic, amino-carboxylic or amino moieties [24] in the alkyl chain bound to the *N*-heterocyclic ring.

Furthermore, since in the case of H_2_(*L2*) and H(*L5*) UV–Vis experiments were carried out in the pH range 2.0–9.1, for these ligands the p*L*_0.5_ values were also calculated at different pHs, *T* = 298.15 K and 310.15 K (Table S3), considering the spectrophotometric data (Table 2).

The sequestering ability of H_2_(*L2*) and H(*L5*) towards Fe^3+^ was found to increase with pH, possibly owing to the gradual ligand deprotonation with pH increasing. The p*L*_0.5_ values also increase with temperature, in accordance with the stability constants trend (Table 2).

### 2.7. Analysis of the pM Values

The study of the metal-chelating affinity of a ligand or the comparison between different ligands’ behaviour towards one or more metal cations can be performed by means of the pM parameter, with pM = −log [M]_free_ (with M = Cu or Fe) for *c*_Mn+_ =1.0·10^−6^ mol L^−1^ and *c*_ligand_ = 1.0·10^−5^ mol L^−1^ [41].

The pM values of all the Cu^2+^ and Fe^3+^/(3,4-HPs) systems investigated in this paper were calculated at pH = 7.4 (physiological value). Furthermore, an attempt to compare the obtained data with those determined for ligands with similar molecular structures and functional groups (Figure S5) was carried out using literature stability constants [22,31,32,33,34,35,36,37,38,39,42], taking into account the already mentioned experimental and methodological differences used for the data treatment.

**Figure 9:**
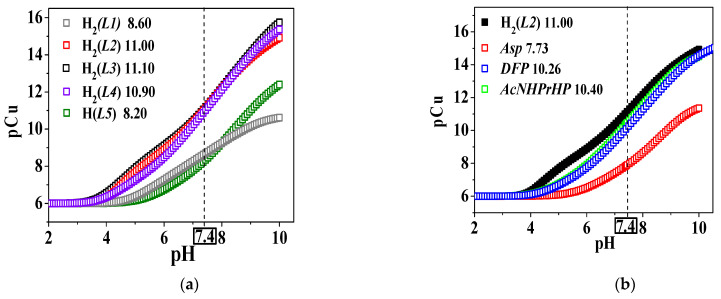
Graphical representation of pCu values calculated vs. pH: (**a**) the (3,4-HPs) under study and (**b**) H_2_*(L2)* and ligands with similar structures. Experimental conditions: *c*_Cu_^2+^ = 1.0·10^−6^ mol L^−1^ and *c*_ligand_ = 1.0·10^−5^ mol L^−1^.

The analysis of the pCu values reported in Table 4 and in Figure 9a showed that, at physiological pH, the copper-chelating affinity by the ligands is favoured by the concomitant presence of -COOH, -NH_2_ and amino-carboxylic groups in the 3,4-HP molecules. They follow the trend: H_2_(*L3*) > H_2_(*L2*) > H_2_(*L4*) > H_2_(*L1*) > H(*L5*). At the selected pH value, an inversion of pCu tendency can be observed for H_2_(*L1*) (terminal -COOH) and H(*L5*) (terminal -NH_2_) with respect to the already mentioned stability constants and sequestration trend. This aspect could be explained considering that at pH = 7.4, the amino group present in H(*L5*) is still protonated while the carboxylic one in H_2_(*L1*) is already deprotonated, thus favouring the Cu^2+^/H_2_(*L1*) electrostatic interaction. At higher pH values, with the deprotonation of -NH_3_^+^ to NH_2_ in H(*L5*), the metal affinity increases with respect to H_2_(*L1*), and the pCu trend becomes analogous to that observed for the stability constants and p*L*_0_._5_ values. A comparison between the pCu data (Table 4, Figure 9b) determined for the Cu^2+^/H_2_(*L2*) system and those calculated for other ligands (Figure S5), such as *DFP* [33,34], *L*-Aspartic acid (*Asp*) [22] and *AcNPrHP* [35], showed that the H_2_(*L2*) copper-chelating affinity at physiological pH and micromolar concentration conditions is higher with respect to the other compounds, following the trend: H_2_(*L2*) > *AcNPrHP > DFP* > *Asp*, with ΔpCu = pCu _H2(*L2*)_–pCu_literature_ = 0.60, 0.74, 3.16, respectively. This trend highlights that the ligands featured by the pyridinone ring display a higher copper affinity with respect to the others, although taking into account some little differences, probably due to the experimental conditions reported in Table 4**.**

The Fe^3+^ chelating efficiency was evaluated at pH = 7.4 only for *L2* and *L5* ligands, since their interaction with the metal cation was also investigated by UV–Vis spectrophotometry, an analytical technique not used for the Fe^3+^/H_2_(*L1*) and Fe^3+^/H_2_(*L4*) systems. In fact, the UV–Vis studies were performed at lower component concentrations (*c* ~ 10^−4^ mol L^−1^) than those used for the potentiometric ones (*c* ~ 10^−3^ mol L^−1^), allowing us to investigate a wider pH range (2.0–9.1) without being stopped at pH ~ 5.0, as occurred for potentiometric titrations, owing to the formation of a precipitate possibly attributable to the formation of the Fe(OH)_3(s)_ species [21].

Analysis of the pFe values, reported in Table 4 for the systems studied herein, showed that at physiological pH the iron-chelating affinity is favoured by the concomitant presence of extra-functional groups in the 3,4-HP ligand molecules, namely the amide-amino-carboxylic moiety (H_2_(*L2*)), with respect to the simple terminal group -NH_2_ (H(*L5*)). The pFe data (Table 4) determined for Fe^3+^/H_2_(*L2*) and Fe^3+^/H(*L5*) systems were also compared with the values reported in the literature for ligands such as H_2_(*L1*) [32], H_2_(*L4*) [31], *DFP* [33,34,36], *Asp* [22], *Orn* [37], H_2_(*S1*) [38], H_2_(*S2*) [39] and H_2_(*S3*) [39]. H_2_(*L2*) and H(*L5*) iron-chelating affinities at pH = 7.4 and micromolar conditions were found to be higher with respect to the literature compounds and follow the trend: H_2_(*L2*) > H(*L5*) > H_2_(*L4*)_Santos_ > *DFP* > H_2_(*S1*) *>* H_2_(*S2*) > H_2_(*S3*) > H_2_(*L1*)_Santos_ > *Orn* ~ *Asp*, with ΔpFe = pFe_H2(*L2*)_–pFe_H(*L5)* or literature_ = 0.40, 2.48, 3.71, 3.79, 4.28, 4.41, 4.48, 8.74, 8.75, respectively. The mentioned trend highlights that the ligands featured by the hydroxo-oxo functionality from the pyridinone ring and amino, amino-carboxylic or amide-amino-carboxylic moieties present a higher metal affinity with respect to the others, even, obviously, taking also into account some little differences, probably due to the experimental conditions reported in Table 4.

### 2.8. Comparison between M^n+^/(3,4-HPs) Systems

**Figure 10:**
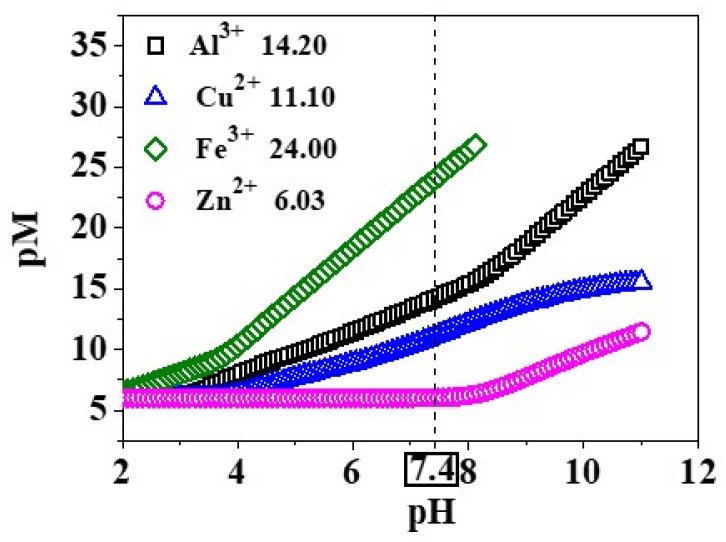
Calculated pM values vs. pH for the different M^n+^/H_2_(*L2*) systems at *T* = 298.15 K, *I* = 0.15 mol L^−1^ in NaCl_(aq)_, *c*_M_^n+^ = 1.0·10^−6^ mol L^−1^ and *c*_ligand_ = 1.0·10^−5^ mol L^−1^.

The data presented in the current paper for Cu^2+^ and Fe^3+^/(3-hydroxy-4-pyridinones) systems (Table 1 and Table 2) were compared with those already reported in the literature on the interaction of the five ligands with Zn^2+^ [19] and Al^3+^ [13] (Table S4) at *I* = 0.15 mol L^−1^ in NaCl_(aq)_ and *T* = 298.15 K. The speciation models determined for the different systems display a common species, namely the M*L*^(n-z)^, which can be used as reference to evaluate and compare the binding ability of the ligands towards the metal cations. The data analysis showed that the log*K*_110_ trend is: Fe^3+^ > Al^3+^ > Cu^2+^ > Zn^2+^, meaning that the 3,4-HPs are featured by a much higher tendency to form very stable complex species with Fe^3+^, followed by Al^3+^, with respect to the M^2+^. In addition, considering the stability constants reported in the literature for the Zn^2+^ [19] and Al^3+^/(H_2_(*L2*)*,* H(*L5*)) systems [13], the pZn and pAl values were also calculated at physiological pH (Table 4) and compared with the analogous results presented in this paper for Cu^2+^ and Fe^3+^. Analysing the data obtained for the different metal cations and the graphs in Figure 10 and Figure S8, we can conclude that, similarly to the log*K*_110_ behaviour, the pM values follow the trend for the metal ions: Fe^3+^ > Al^3+^ > Cu^2+^ > Zn^2+^. Thus, both of the 3-hydroxy-4-pyridinones present a higher chelating affinity towards Fe^3+^, and in a lesser extent also to Al^3+^, with respect to divalent metal cations.

These trends could probably be justified taking into account the already mentioned “*hard*-*soft* acids and bases” theory (HSAB) [27,28,29], according to which a *hard* acid–*hard* base or a *soft* acid–*soft*-base interactions are kinetically and thermodynamically favoured if compared with *hard*–*soft* ones. On this basis, the affinity between *hard* metal cations (acids: Fe^3+^, Al^3+^) and *hard*-base functional groups (bases: -OH, -COOH) is higher with respect to those with *borderline* acids like Cu^2+^ and Zn^2+^.

In light of these considerations, it can be claimed that, from a thermodynamic point of view, most of the bifunctional 3,4-HP ligands studied herein are particularly selective towards Fe^3+^ and could be considered promising iron-chelating agents, also avoiding the possibility of a significant competition, and eventually a depletion, of divalent metals with biological and environmental relevance, such as Cu^2+^ and Zn^2+^.

## 3. Materials and Methods

### 3.1. Chemicals

Riedel–deHäen concentrated ampoules were used to prepare sodium hydroxide and hydrochloride solutions standardized against potassium hydrogen phthalate and sodium carbonate, respectively. NaOH solutions were preserved from atmospheric carbon dioxide by means of soda lime traps. CuCl_2_·2H_2_O and FeCl_3_·6H_2_O salts purchased by Fluka were weighed to prepare the metal solutions without further purification and standardized against EDTA standard solutions [43]; their purity was always ≥98%. The synthesis of the functionalized 3-hydroxy-4-pyridinones was already reported in the literature [13]. The ligand solutions were prepared by weighing the products in the neutral form (H_r_(*L*)^0^) without any further purification. Their purity was checked by means of alkalimetric measurements and, for all the ligands, it was found to be ≥99.5%. The ionic medium aqueous solutions were prepared by weighing the pure Fluka NaCl salt, previously dried in an oven at *T* = 383.15 K for two hours. The reagents used to carry out the studies were of the best available purity. The preparation of the solutions was performed using analytical grade water (R = 18 MΩ cm^−1^) and grade A glassware.

### 3.2. Analytical Instrumentation and Procedures

#### 3.2.1. Potentiometric Equipment and Procedure

The interactions of the five 3-hydroxy-4-pyridinones towards Cu^2+^ and Fe^3+^ were experimentally investigated using a Metrohm 809 Titrando and a potentiometer with a combined Thermo-Orion glass electrode (Ross type 8102) connected to an automatic burette. This apparatus was coupled to a personal computer, and automatic titrations were performed by means of the MetrohmTiAMO 1.2 software, useful for the control of titrant delivery, data acquisition and e.m.f. stability. The estimated accuracy, for e.m.f. and titrant volume readings, was ±0.15 mV and ±0.003 mL, respectively. The measurements were carried out in 25 mL thermostatted cells under magnetic stirring, and purified presaturated nitrogen was bubbled into the solutions for at least 5 min to exclude the presence of oxygen and carbon dioxide inside. For all the experiments, titrations of hydrochloric acid with standard NaOH solutions were carried out at the same temperature, ionic strength and ionic medium conditions with respect to those used for the systems under study, for refining the value of the electrode potential (E^0^), the acidic junction potential (Ej = j_a_[H^+^]) and the ionic product of water (*K*_w_). The pH scale employed was the free scale and pH≡ −log[H^+^], with [H^+^] that is the free concentration of the proton. From sixty to one hundred data points were collected during each titration, depending on the possible formation of sparingly soluble species.

The potentiometric titrations were carried out at *I* = 0.15 mol L^−1^ in NaCl_(aq)_, *T* = 298.15 K and different concentrations of ligands (*c*_ligand_ = 5.0·10^−4^–1.5·10^−3^ mol L^−1^) and metal cations (*c*_Mn+_ = 4.3·10^−4^–1.0·10^−3^ mol L^−1^). The pH ranges investigated were 2.0–10.0 and 2.0–5.0 for Cu^2+^ and Fe^3+^/(3,4-HPs) measurements, respectively, due to the formation of sparingly soluble species.

#### 3.2.2. UV–Vis Spectrophotometric Apparatus and Procedure

The UV–Vis spectrophotometric titrations were carried out using a Varian Cary 50 spectrophotometer presenting an optic fibre probe with a fixed 1-cm path length. This instrument was connected to a computer, and the recording of absorbance (A) signal vs. wavelength (λ / nm) was carried out by means of the Varian Cary WinUV software. At the same time, a Thermo-Orion combined glass electrode (Ross type 8102), linked to a potentiometer, was employed to collect potentiometric data. The NaOH titrant solution was delivered in a 25-mL titration cell using an automatic burette (Metrohm 665 model). The homogeneity of the solutions during the measurements was ensured using a magnetic stirrer. Nitrogen was bubbled in the solutions for at least 5 min before starting the experiments, also in this case, for excluding the presence of O_2(g)_ and CO_2(g)_ inside.

The binding ability of H_2_(*L2*), H_2_(*L4*) and H(*L5*) towards Cu^2+^ and Fe^3+^ was also studied by means of UV–Vis spectrophotometric titrations of solutions containing different concentrations of ligands (*c*_ligand_ = 1.0·10^−5^–2.1·10^−4^ mol L^−1^) and metal cations (*c*_Mn+_ = 5.0·10^−6^–1.5·10^−4^ mol L^−1^). The experiments were performed at *I =* 0.15 mol L^−1^ in NaCl_(aq)_, *T* = 298.15 K, a wavelength of 200 ≤ λ / nm ≤ 800, pH ranges 2.0–11.0 and 2.0–9.1 for Cu^2+^ and Fe^3+^/(3,4-HPs) investigations, respectively. In the case of the Fe^3+^/H_2_(*L2*) and Fe^3+^/H(*L5*) systems, the measurements were also carried out at *I* = 0.15 mol L^−1^ in NaCl_(aq)_ and *T* = 310.15 K.

### 3.3. Computer Programmes

Appropriate computer programmes were employed for the treatment of experimental data from different analytical techniques. The non-linear least squares ESAB2M computer program [44] was used for the determination of the acid–base titrations parameters (E^0^, pK_w_, j_a_) and the reagents’ analytical concentration. The elaboration of potentiometric data was carried out by means of the BSTAC computer program [45], while the UV–Vis spectrophotometric ones were processed using the HypSpec 2008 [46]. The calculation of the M^n+^/3-hydroxy-4-pyridinone species formation percentages and the representation of distribution diagrams was performed using the HySS program [30].

## 4. Conclusions

The binding ability of five bifunctional 3-hydroxy-4-pyridinones towards Cu^2+^ and Fe^3+^ was studied by means of potentiometric and UV–Vis spectrophotometric measurements carried out at *I* = 0.15 mol L^−1^ in NaCl_(aq)_ and *T* = 298.15 K. The data treatments allowed us to determine the speciation schemes featured by metal-ligand species with different stoichiometry and stability, due to the various functional groups present in the 3-hydroxy-4-pyridinones structures, which could potentially participate in the metal complexation and in the Cu^2+^ and Fe^3+^ behaviour in an aqueous solution. The stability of metal-ligand species follows the trends: Cu(*L3*)^0^_(aq)_ > Cu(*L4*)^0^_(aq)_ > Cu(*L2*)^0^_(aq)_ > Cu(*L5*)^+^ > Cu(*L1*)^0^_(aq)_ and: Fe(*L4*)^+^ > Fe(*L2*)^+^ > Fe(*L5*)^2+^ > Fe(*L1*)^+^, respectively. They were favoured by the simultaneous presence of amino or amino-carboxylic bearing groups in the 3,4-HP ligands, and showed some dependence on the length and structure of the chains between the pyridinone ring and the extra-functional groups. The investigation of the sequestering ability and metal-chelating efficiency was carried out by the calculation of the p*L*_0_._5_ and pM parameters at different pHs and physiological value (pH = 7.4), respectively. Similarly to the complexation behaviour, the sequestration and Cu^2+^ and Fe^3+^ affinity by the ligands under study is affected by the presence in the whole 3-hydroxy-4-pyridinone molecules of terminal amino-carboxylic groups and amidic moiety in the alkyl chain or, at least, of the one single terminal group, as -NH_2_ group (H(*L5*)), with respect to the carboxylic group (H_2_(*L1*)). In addition, the data presented in this paper for Cu^2+^ and Fe^3+^/3-hydroxy-4-pyridinone systems were compared with those reported in the literature, for the interaction of the ligands with Al^3+^ and Zn^2+^ at *I* = 0.15 mol L^−1^ in NaCl_(aq)_ and *T* = 298.15 K. The log*K*_110_ and pM trend show a clear dependence on the metal ion (Fe^3+^ > Al^3+^ > Cu^2+^ > Zn^2+^), meaning that the 3-hydroxy-4-pyridinones display a higher stability and chelating affinity towards Fe^3+^ and (in a lesser degree) also Al^3+^, with respect to divalent metal cations. In light of these considerations, it can be claimed that, from a thermodynamic point of view, the ligands are particularly selective towards Fe^3+^ and could be considered promising iron-chelating agents, also avoiding the possibility of a significant competition, and eventually a depletion, of divalent metals with biological and environmental relevance, such as Zn^2+^ and Cu^2+^.

## Figures and Tables

**Table 1:** Experimental stability constants ^1^ of Cu^2+^/3-hydroxy-4-pyridinone species obtained by different analytical techniques at *I* = 0.15 mol L^−1^ in NaCl_(aq)_, *T* = 298.15 K and *p* = 0.1 MPa.

	log*β*_pqr_ (log*K*_pqr_)		
Species	Potentiometry	UV–Vis Spectrophotometry	Average Stability Constants ^2^
CuH(*L1*)^+^	14.23 ± 0.04 ^3^ (4.28)	-	-
Cu(*L1*)^0^_(aq)_	9.76 ± 0.06	-	-
CuH_2_(*L2*)^2+^	24.98 ± 0.02 ^3^ (5.46)	24.93 ± 0.05 ^3^ (5.41)	24.95 ± 0.07 ^4^ (5.43)
CuH(*L2*)^+^	21.75 ± 0.03 (11.02)	21.00 ± 0.04 (10.27)	21.37 ± 0.30 (10.64)
Cu(*L2*)^0^_(aq)_	15.10 ± 0.10	14.48 ± 0.105	14.79 ± 0.26
CuH_2_(*L3*)^2+^	26.93 ± 0.02 ^3^ (6.26)	-	-
Cu(*L3*)H^+^	22.84 ± 0.03 (11.85)	-	-
Cu(*L3*)^0^_(aq)_	15.95 ± 0.03	-	-
CuH_2_(*L4*)^2+^	25.65 ± 0.02 ^3^ (5.21)	25.66 ± 0.04 ^3^ (5.22)	25.65 ± 0.01 ^4^ (5.21)
CuH(*L4*)^+^	21.63 ± 0.03 (10.53)	21.78 ± 0.02 (10.68)	21.70 ± 0.04 (10.60)
Cu(*L4*)^0^_(aq)_	15.62 ± 0.07	15.62 ± 0.05	15.62 ± 0.06
CuH(*L5*)^2+^	20.00 ± 0.03 ^3^ (8.92)	20.241 ± 0.006 ^3^ (9.161)	20.12 ± 0.09 ^4^ (9.04)
Cu(*L5*)^+^	12.62 ± 0.06	12.67 ± 0.04	12.64 ± 0.04

^1^ log*β*_pqr_ and log*K*_pqr_ refer to Equations (2) and (1), respectively; ^2^ data obtained by an average of potentiometric and UV–Vis spectrophotometric data; ^3^ ±Std. Dev.; ^4^ errors on weighed data. Standard uncertainties: u(*T*) = 0.1 K; u(*I*) = 0.01 mol L^−1^.

**Table 2:** Experimental stability constants ^1^ of Fe^3+^/(3,4-HP) species obtained by different analytical techniques at *I* = 0.15 mol L^−1^ in NaCl_(aq)_, different temperatures and *p* = 0.1 MPa.

	log*β*_pqr_ (log*K*_pqr_)			
	*T* = 298.15 K			*T* = 310.15 K
Species	Potentiometry	UV–Vis Spectrophotometry	Average Stability Constants ^2^	UV–Vis Spectrophotometry
FeH(*L1*)^2+^	17.37 ± 0.10 ^3^(7.42)	-	-	-
Fe(*L1*)^+^	13.23 ± 0.19	-	-	-
Fe(*L1*)_2_^-^	22.52 ± 0.20 (9.29)	-	-	-
FeH(*L2*)^2+^	26.16 ± 0.03 ^3^(15.43)	25.91 ± 0.04^3^(15.18)	26.03 ± 0.10 ^4^(15.30)	26.23 ± 0.01 ^3^(15.24)
Fe(*L2*)^+^	22.06 ± 0.06	21.78 ± 0.03	21.97 ± 0.11	22.86 ± 0.04
Fe(*L2*)_2_^-^	38.01 ± 0.04 (15.95)	38.01 ^5^ (16.23)	38.01 ± 0.11(16.04)	39.79 ± 0.08 (16.83)
FeH(*L4*)^2+^	26.31 ± 0.02 ^3^(15.21)	-	-	-
Fe(*L4*)^+^	22.48 ± 0.03	-	-	-
Fe(*L4*)_2_^-^	39.08±0.03 (16.60)	-	-	-
FeH(*L5*)^3+^	25.05 ± 0.04 ^3^(13.97)	24.85 ± 0.04 ^3^(13.77)	24.95±0.15 ^4^ (13.87)	25.35 ± 0.04 ^3^(14.75)
Fe(*L5*)^2+^	20.93 ± 0.03	20.30 ± 0.03	20.61±0.27	21.16 ± 0.08

^1^ log*β*_pqr_ and log*K*_pqr_ refer to Equations (2) and (1), respectively; ^2^ values obtained by an average of potentiometric and UV–Vis spectrophotometric data; ^3^ ±Std. Dev.; ^4^ errors on weighed data; ^5^ value kept constant from potentiometric data. Standard uncertainties: u(*T*) = 0.1 K; u(*I*) = 0.01 mol L^−1^.

**Table 3:** p*L*_0.5_^1^ values of Cu^2+^/ligands systems at different pHs, *I* = 0.15 mol L^−1^ in NaCl_(aq)_ and *T* = 298.15 K.

Ligand	pH	p*L*_0.5_
H_2_*(L1)*	7.4	7.09
H_2_*(L2)*	2.5	2.74
	3.0	3.70
	4.0	5.48
	5.0	6.99
	6.0	8.18
	7.4	10.29
	8.1	10.54
	9.0	11.98
H_2_*(L3)*	2.5	3.10
	3.0	4.05
	4.0	5.70
	5.0	7.18
	6.0	8.41
	7.4	10.30
	8.1	11.43
	9.0	12.20
	10.0	12.14
H_2_*(L4)*	7.4	9.90
H*(L5)*	7.4	7.25

^1^ values calculated by Equation (3).

**Table 4:** pM values calculated for different Cu^2+^ and Fe^3+^/(3,4-HPs) systems based on 3-hydroxy-4-pyridinone ligands and similar structures’ ligands at pH = 7.4 from stability constants reported in the literature.

M^n+^	Ligand	pM	Ref.	M^n+^	Ligand	pM	Ref.
Cu^2+^	H_2_*(L1)*	8.60	This work	Fe^3+^	H_2_*(L4)*	21.90 ^3^	[31]
Cu^2+^	H_2_*(L2)*	11.00	This work	Fe^3+^	Deferiprone	20.70 ^1^	[34]
Cu^2+^	H_2_*(L3)*	11.10	This work	Fe^3+^	H_2_*(S1)*	20.59 ^1^	[38]
Cu^2+^	H_2_*(L4)*	10.90	This work	Fe^3+^	H_2_*(S2)*	20.19 ^6^	[39]
Cu^2+^	H_2_*(L5)*	8.20	This work	Fe^3+^	H_2_*(S3)*	19.97 ^6^	[39]
Cu^2+^	Deferiprone	10.69 ^1^	[34]	Fe^3+^	*L*-Aspartic acid	15.63 ^4^	[22]
Cu^2+^	*L*-Aspartic acid	7.84^2^	[22]	Fe^3+^	*L*-Ornithine	15.64 ^5^	[37]
Cu^2+^	*AcNPrHP*	10.40 ^1^	[35]	Al^3+^	H_2_*(L2)*	14.20 ^2^	[13]
Fe^3+^	H_2_*(L2)*	24.38	This work	Al^3+^	H_2_*(L5)*	13.17 ^2^	[13]
Fe^3+^	H_2_*(L5)*	23.98	This work	Zn^2+^	H_2_*(L2)*	6.03 ^2^	[19]
Fe^3+^	H_2_*(L1)*	19.90 ^3^	[32]	Zn^2+^	H_2_*(L5)*	8.28 ^2^	[19]

^1^*I* = 0.10 mol L^−1^ in KCl_(aq)_, *T* = 298.15 K; ^2^ *I* = 0.15 mol L^−1^ in NaCl_(aq)_, *T* = 298.15 K; ^3^ *I* = 0.10 mol L^−1^ in KNO_3(aq)_, *T* = 298.15 K; ^4^ *I* = 1.00 mol L^−1^ in Na^+^ ionic medium, *T* = 293.15 K; ^5^ *I* = 0.10 mol L^−1^ in KNO_3(aq)_, *T* = 293.15 K; ^6^ *I* = 0.10 mol L^−1^ in MOPS (3-(*N*-morpholino)propanesulphonic acid) buffer at pH = 7.4, *T* = 298.15 K.

## Data Availability

All the experimental data are reported in the main text or in supporting files. Any other information about data handling may be obtained upon contacting Anna Irto (airto@unime.it).

## Supplementary Materials

The following are available online, Table S1. Overall and stepwise protonation constants of the 3-hydroxy-4-pyridinones under study reported in the literature at *I* = 0.15 mol L^−1^ in NaCl_(aq)_ and different temperatures; Table S2. Literature stability constants of Cu^2+^ and Fe^3+^/ligand species reported at different temperatures, ionic strengths and ionic media in molar concentration scale; Table S3. p*L*_0.5_ values of Fe^3^^+^/H_2_(*L**2*) and H(*L5*) systems at different pHs and temperature, from UV–Vis data at *I* = 0.15 mol L^−1^ in NaCl_(aq)_; Table S4. Literature stability constants of Zn*L*^(2-z)^ and Al*L*^(3-z)^ species at *I* = 0.15 mol L^−1^ in NaCl_(aq)_, *T* = 298.15 K; Figure S1. Distribution diagram of Cu^2+^/(3,4-HPs) systems at *T* = 298.15 K, *I* = 0.15 mol L^−1^ in NaCl_(aq)_, *c*_Cu_^2+^ = 5.0·10^−4^ mol L^−1^ and *c*_ligand_ = 1.5·10^−3^ mol L^−1^. Ligands = H_2_(*L**1*) (a), H_2_(*L**3*) (b); H_2_(*L**4*) (c), H(*L5*) (d); Figure S2. Distribution diagram of Fe^3+^/(3,4-HPs) systems at *T* = 298.15 K, *I* = 0.15 mol L^−1^ in NaCl_(aq)_, *c*_Fe_^3+^ = 5.0·10^−4^ mol L^−1^ and *c*_ligand_ = 1.1·10^−3^ mol L^−1^. Ligands = H_2_(*L**1*) (a), H_2_(*L**4*) (b), H(*L5*) (c); Figure S3. UV–Vis absorption profile Fe^3+^/H_2_(*L**2*) system at *I* = 0.15 mol L^−1^ in NaCl_(aq)_, *T* = 310.15 K and at different pH values. (a) *c*_Fe3+_ = 2.3·10^−4^ mol L^−1^, *c*_ligand_ = 2.2·10^−4^ mol L^−1^; (b) *c*_Fe3+_ = 2.0·10^−4^ mol L^−1^, *c*_ligand_ = 4.0·10^−4^ mol L^−1^; Figure S4. UV–Vis absorption profile Fe^3+^/H(*L5*) system at *I* = 0.15 mol L^−1^ in NaCl_(aq)_, different temperatures and pH values. (a) *T* = 298.15 K, *c*_Fe3+_ = 2.4·10^−4^ mol L^−1^, *c*_ligand_ = 2.6·10^−4^ mol L^−1^; (b) *T* = 310.15 K, *c*_Fe3+_ = 2.5·10^−4^ mol L^−1^, *c*_ligand_ = 2.4·10^−4^ mol L^−1^; Figure S5. Graphical representation of molar absorptivity of Fe^3+^/H_2_(*L**2*) species at *T* = 298.15 K (a) and 310.15 K (b), *I* = 0.15 mol L^−1^ in NaCl_(aq)_; Figure S6. Molecular structures of compounds with similar structures and functional groups with respect to 3-hydroxy-4-pyridinones; Figure S7. Sequestration diagrams of: (a) Cu^2+^/H_2_(*L**2*) species at *I* = 0.15 mol L^−1^ in NaCl_(aq)_ and *T* = 298.15 K and different pHs, p*L*_0.5_ values: 2.74 (pH = 2.5), 3.70 (pH = 3.0), 5.48 (pH = 4.0), 6.99 (pH = 5.0), 8.18 (pH = 6.0), 10.29 (pH = 7.4), 10.54 (pH = 8.1), 11.98 (pH = 9.0); (b) Cu^2+^/(3,4-HPs) systems at the same ionic strengths and temperature, pH = 7.4. p*L*_0.5_ values: 7.09 (H_2_(*L**1*)), 10.29 (H_2_(*L**2*)), 10.30 (H_2_(*L**3*))*,* 9.90 (H_2_(*L**4*)), 7.25 (H(*L5*)); Figure S8. Calculated pM values vs. pH for the different M^n+^/*L5* systems at *T* = 298.15 K, *I* = 0.15 mol L^−1^ in NaCl_(aq)_, *c*_M_^n+^ = 1.0·10^−6^ mol L^−1^ and *c*_ligand_ = 1.0·10^−5^ mol L^−1^.
