# Supplementary material for: Bifunctional 3-Hydroxy-4-Pyridinones as Potential Selective Iron(III) Chelators: Solution Studies and Comparison with Other Metals of Biological and Environmental Relevance
Source: Molecules. 2021 Nov 30;26(23):7280. doi: 10.3390/molecules26237280 (PMC8658793; doi:10.3390/molecules26237280)
Supplement: Supplementary file 1 [file molecules-26-07280-s001.zip › molecules-1459761-supplementary.pdf]

SUPPLEMENTARY MATERIALS

Bifunctional 3-hydroxy-4-pyridinones as potential selective iron(III) chelators: solution studies and comparison with other metals of biological and environmental relevance

Anna Irto,*[1] Paola Cardiano,[1] Karam Chand,[2] Rosalia Maria Cigala,[1] Francesco Crea,[1] Concetta De Stefano[1] and Maria Amélia Santos*[2]

[1] *Dipartimento di Scienze Chimiche, Biologiche, Farmaceutiche e Ambientali, Università di Messina, Viale F. Stagno d'Alcontres, 31 – 98166 Messina, Italy*

[2] *Centro de Química Estrutural, Instituto Superior Técnico, Universidade de Lisboa, Av. Rovísco Pais 1, 1049-001 Lisboa, Portugal*

*Corresponding authors: Anna Irto, email: airto@unime.it; Maria Amélia Santos, email: masantos@tecnico.ulisboa.pt

Tables

Table S1. Overall and stepwise protonation constants[1] of the 3-hydroxy-4-pyridinones under study reported in the literature at I = 0.15 mol L^{-1} in $NaCl_{(aq)}$ and different temperatures [13].

Ligand	I/mol L^{-1}	T/K	$\log K_1^H$	$\log\beta_2^H$ ($\log K_2^H$)	$\log\beta_3^H$ ($\log K_3^H$)	$\log\beta_4^H$ ($\log K_4^H$)
$H_2(L1)$	0.15	298.15	9.947	14.36 (4.41)	17.74 (3.38)	-
$H_2(L2)$	0.15	298.15	10.73	19.52 (8.79)	24.17 (4.65)	27.43 (3.26)
	0.15	310.15	10.99	17.05 (6.06)	21.02 (3.97)	24.08 (3.06)
$H_2(L3)$	0.15	298.15	10.93	20.70 (9.77)	25.60 (4.90)	29.02 (3.42)
$H_2(L4)$	0.15	298.15	11.10	20.44 (9.34)	24.60 (4.16)	27.87 (3.27)
$H(L5)$	0.15	298.15	11.08	20.468(9.388)	23.68 (3.21)	-
	0.15	310.15	10.57	16.53 (5.96)	19.53 (3.00)	-

[1] $\log\beta_r^H$ and $\log K_r^H$ refer to the equilibria: $rH^+ + L^{z-} = H_rL^{-(z-r)}$ and $H^+ + H_{(r-1)}L^{-(z-(r-1))} = H_rL^{-(z-r)}$, respectively.

Table S2. Literature stability constants of Cu^{2+} and Fe^{3+}/ligands species reported at different temperatures, ionic strengths and ionic media in molar concentration scale

M^{n+}	Ligand	$\log\beta_{MH_2L}$	$\log\beta_{MHL}$	$\log\beta_{ML}$	$\log\beta_{ML_2}$	$\log\beta_{MHL_2}$	$\log\beta_{MH_2L_2}$	$\log\beta_{MOHL_2}$	$\log\beta_{ML_3}$	$\log\beta_{MHL_3}$	$\log\beta_{MH_2L_3}$	$\log\beta_{MH_3L_3}$	$\log\beta_{M_2L}$	$\log\beta_{M_2L_2}$	$\log\beta_{M_2L_3}$	Ref.
Cu^{2+}	*DFP*[1]	-	-	10.62	19.68	-	-	-	-	-	-	-	-	-	-	[33]
Cu^{2+}	*DFP*[1]	-	-	10.42	19.09	21.98	-	8.49	-	-	-	-	-	-	-	[34]
Cu^{2+}	*Asp*[2]	-	12.84	9.04	15.86	-	-	-	-	-	-	-	-	-	-	[22]
Cu^{2+}	*AcNPrHP*[1]	-	-	10.29	18.99	-	-	-	-	-	-	-	-	-	-	[35]
Fe^{3+}	H_2(*L1*)[3]	-	19.62	-	28.79	33.12	36.45	-	37.01	-	-	-	-	-	-	[32]
Fe^{3+}	H_2(*L4*)[3]	-	25.01	-	-	-	46.20	-	-	48.43	57.52	65.68	-	-	-	[31]
Fe^{3+}	*DFP*[1]	-	-	15.10	26.61	-	-	-	35.88	-	-	-	-	-	-	[33]
Fe^{3+}	*DFP*[1]	-	-	15.14	26.68	-	-	-	35.92	-	-	-	-	-	-	[36]
Fe^{3+}	*DFP*[1]	-	-	15.01	27.30	-	-	-	37.43	-	-	-	-	-	-	[34]
Fe^{3+}	*DFP*[1]	-	-	14.80	26.70	-	-	-	36.56	-	-	-	-	-	-	[34]
Fe^{3+}	H_2(*S1*)[1]	-	-	14.26	25.73	34.91	-	-	-	-	-	-	-	-	-	[38]
Fe^{3+}	H_2(*S2*)[5]	-	-	15.70	27.30	36.70	-	-	-	-	-	-	-	-	-	[39]
Fe^{3+}	H_2(*S3*)[5]	-	-	16.60	28.00	35.90	-	-	-	-	-	-	-	-	-	[39]
Fe^{3+}	*Asp*[6]	-	-	11.40	-	-	-	-	-	-	-	-	-	-	-	[22]
Fe^{3+}	*Orn*[7]	-	-	8.70	-	-	-	-	-	-	-	-	-	-	-	[37]

[1] I = 0.10 mol L^{-1} in $KCl_{(aq)}$, T = 298.15 K; [2] I = 0.15 mol L^{-1} in $NaCl_{(aq)}$, T = 298.15 K; [3] I = 0.10 mol L^{-1} in $KNO_{3(aq)}$, T = 298.15 K; [4] I = 0.10 mol L^{-1} in $KCl_{(aq)}$, T = 310.15 K; [5] I = 0.10 mol L^{-1} in MOPS (3-(*N*-morpholino)propanesulphonic acid) buffer at pH = 7.4, T = 298.15 K; [6] I = 0.10 mol L^{-1} in Na^+ background electrolyte, T = 293.15 K; [7] I = 0.10 mol L^{-1} in $NaClO_{4(aq)}$, T = 293.15 K.

Ligands abbreviations: *DFP* = Deferiprone; *Asp* = *L*-Aspartic acid; *Orn* = *L*-Ornithine; *AcNPrHP* = 1-(3′-methylcarboxyaminopropyl)-3-hydroxy-2-methyl-4-pyridinone; H_2(*L1*) = 4-(3-hydroxy-2-methyl-4-oxopyridin-1(4H)-yl)butanoic acid; H_2(*L4*) = (S)-2-amino-5-(3-hydroxy-2-methyl-4-oxopyridin-1(4H)-yl)pentanoic acid; H_2(*S1*) = 3-hydroxy-2-methylpyridin-4(1H)-one; H_2(*S2*) = 3-hydroxy-1-(2-hydroxyethyl)-2-methylpyridin-4(1H)-one; H_2(*S3*) = 3-(3-hydroxy-2-methyl-4-oxopyridin-1(4H)-yl)propanoic acid.

Table S3. $pL_{0.5}$[1] values of $Fe^{3+}/H_2(L2)$ and $Fe^{3+}/H(L5)$ systems at different pHs and temperature, from UV-Vis data at I = 0.15 mol L^{-1} in $NaCl_{(aq)}$

Ligand	T/K	pH	$pL_{0.5}$	Ligand	T/K	pH	$pL_{0.5}$
$H_2(L2)$	298.15	2.0	4.45	$H_2(L2)$	310.15	5.0	11.37
$H_2(L2)$	298.15	3.0	6.88	$H_2(L2)$	310.15	6.0	11.42
$H_2(L2)$	298.15	4.0	8.12	$H_2(L2)$	310.15	7.4	11.35
$H_2(L2)$	298.15	5.0	8.60	$H(L5)$	310.15	2.0	10.41
$H_2(L2)$	298.15	6.0	8.77	$H(L5)$	310.15	3.0	10.83
$H_2(L2)$	298.15	7.4	9.45	$H(L5)$	310.15	4.0	10.92
$H_2(L2)$	298.15	8.1	9.83	$H(L5)$	310.15	5.0	11.37
$H_2(L2)$	310.15	2.0	8.04	$H(L5)$	310.15	6.0	11.52
$H_2(L2)$	310.15	3.0	10.36	$H(L5)$	310.15	7.4	11.51
$H_2(L2)$	310.15	4.0	11.26				

[1] values calculated by eq. (3).

Table S4. Literature stability constants of Zn$L^{(2-z)}$ [19] and Al$L^{(3-z)}$ [13] species at I = 0.15 mol L^{-1} in $NaCl_{(aq)}$, T = 298.15 K.

Ligand	$\log K_{110}$[1]	
	Zn$L^{(2-z)}$	Al$L^{(3-z)}$
H_2(*L1*)	7.27	12.57
H_2(*L2*)	8.20	17.90
H_2(*L3*)	9.52	17.57
H_2(*L4*)	9.68	18.60
H(*L5*)	9.22	15.08

[1] $\log K_{110}$ values refer to equilibrium: $M^{n+} + L^{z-} = ML^{(n-z)}$, where $M^{n+} = Zn^{2+}$ and Al^{3+}

Figures

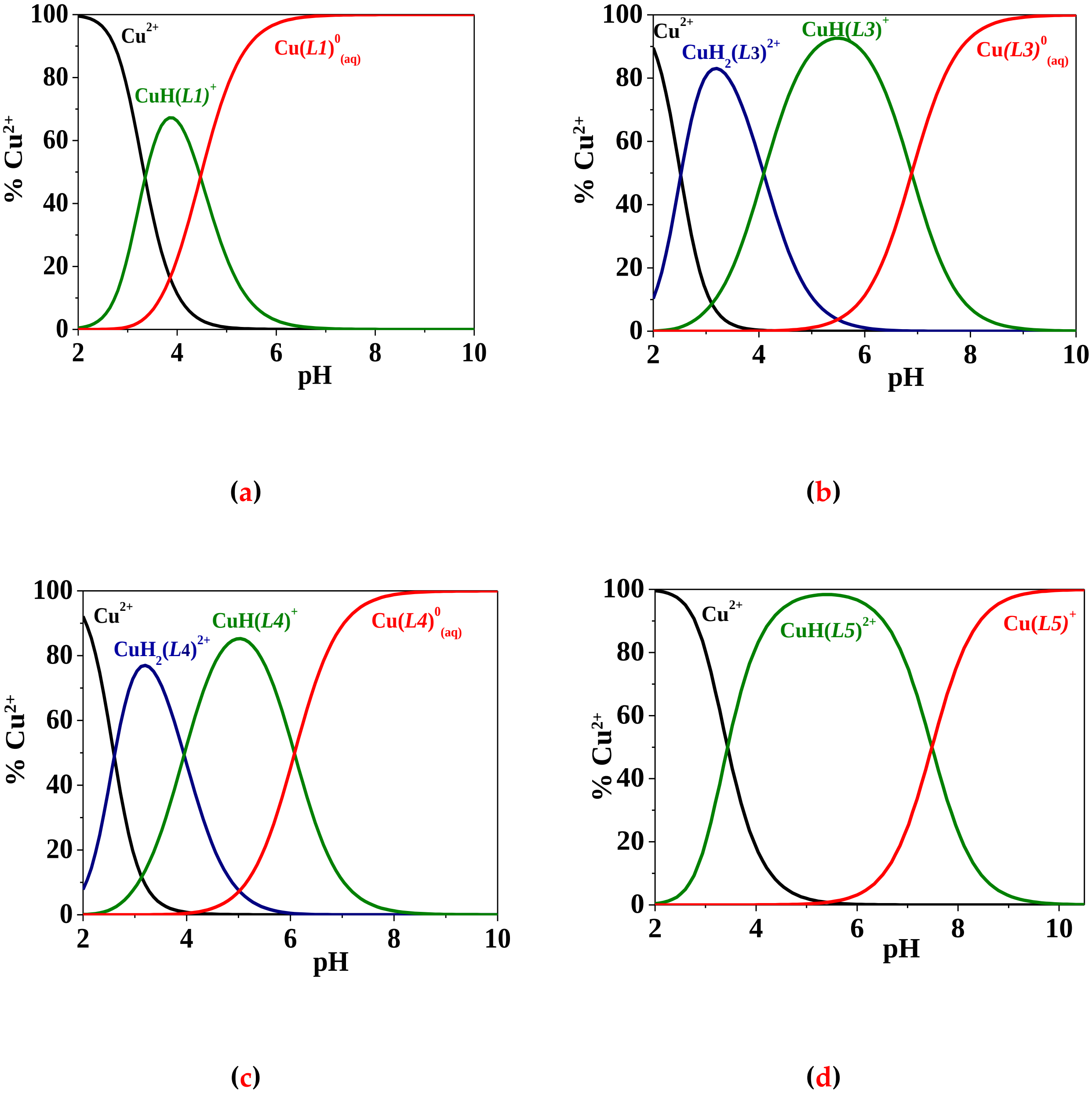

Figure S1. Distribution diagram of Cu^{2+}/(3,4-HPs) systems at T = 298.15 K, I = 0.15 mol L^{-1} in $NaCl_{(aq)}$, $c_{Cu^{2+}}$ = $5.0 \cdot 10^{-4}$ mol L^{-1} and c_{ligand} = $1.5 \cdot 10^{-3}$ mol L^{-1}. Ligands = H_2(*L1*) **(a)**, H_2(*L3*) **(b)**, H_2(*L4*) **(c)**, H(*L5*) **(d)**.

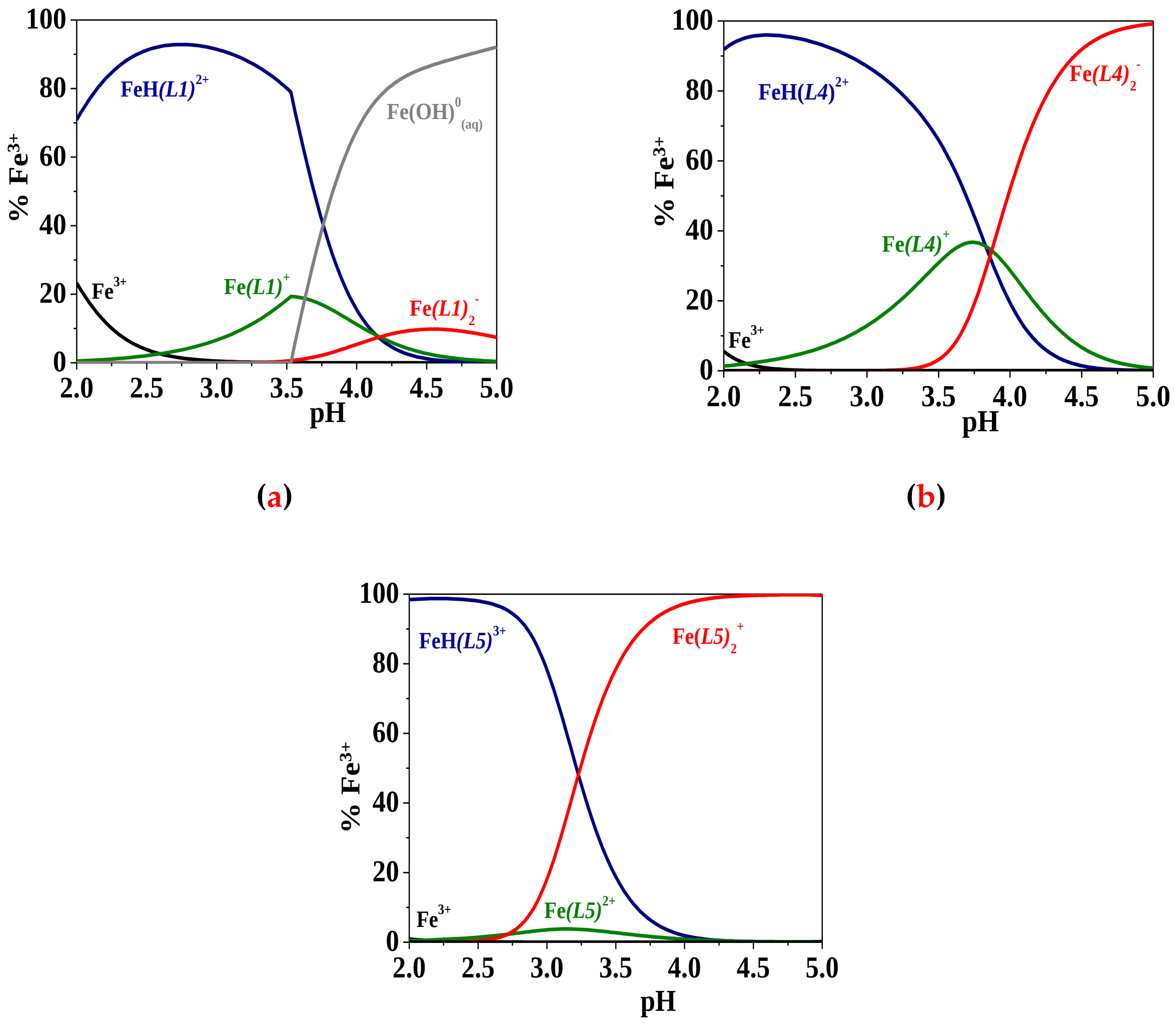

(a) (b) (c)

Figure S2. Distribution diagram of Fe^{3+}/(3,4-HPs) systems at T = 298.15 K, I = 0.15 mol L^{-1} in $NaCl_{(aq)}$, $c_{Fe^{3+}}$ = $5.0\cdot10^{-4}$ mol L^{-1} and c_{ligand} = $1.1\cdot10^{-3}$ mol L^{-1}. Ligands = H_2(*L1*) **(a)**, H_2(*L4*) **(b)**, H(*L5*) **(c)**.

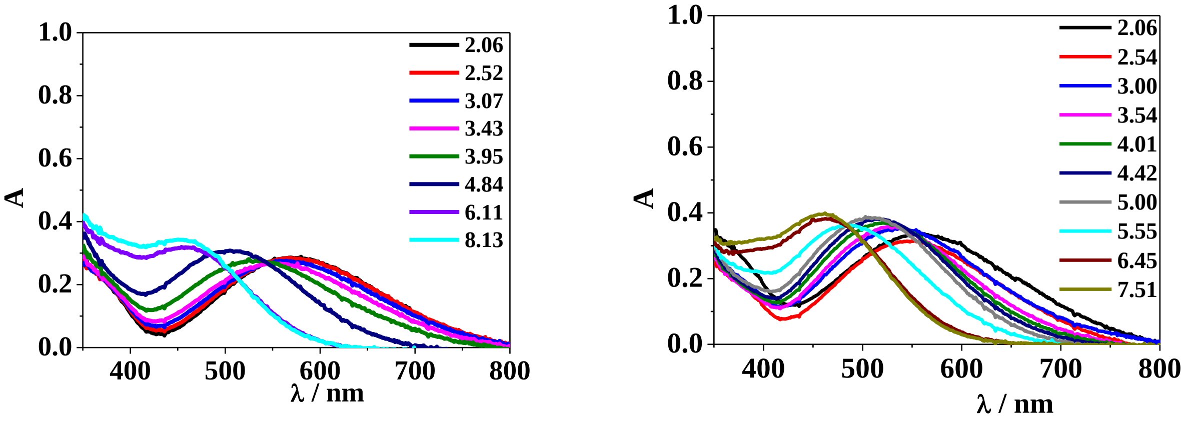

(a) **(b)**

Figure S3. UV-Vis absorption profile of Fe^{3+}/H_2(*L2*) system at I = 0.15 mol L^{-1} in $NaCl_{(aq)}$, T = 310.15 K and at different pH values.

(a) $c_{Fe^{3+}} = 2.3 \cdot 10^{-4}$ mol L^{-1}, $c_{ligand} = 2.2 \cdot 10^{-4}$ mol L^{-1};

(b) $c_{Fe^{3+}} = 2.0 \cdot 10^{-4}$ mol L^{-1}, $c_{ligand} = 4.0 \cdot 10^{-4}$ mol L^{-1}.

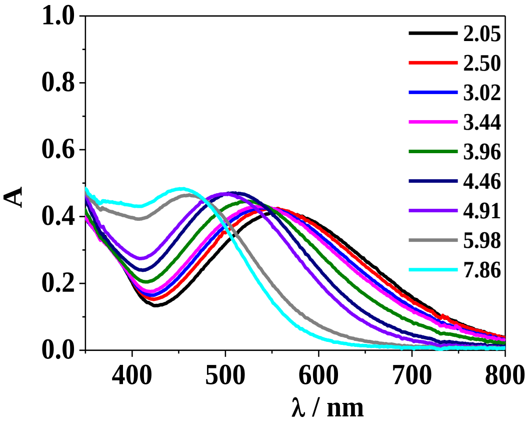

(**a**)

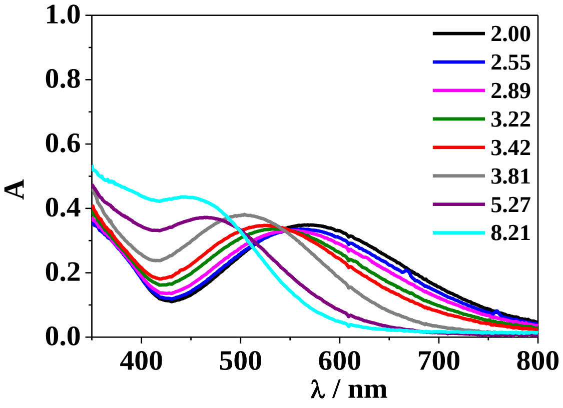

(**b**)

Figure S4. UV-Vis absorption profile of Fe^{3+}/H(*L5*) system at I = 0.15 mol L^{-1} in $NaCl_{(aq)}$, different temperatures and pH values.

(**a**) T = 298.15 K, $c_{Fe^{3+}}$ = 2.4·10^{-4} mol L^{-1}, c_{ligand} = 2.6·10^{-4} mol L^{-1};

(**b**) T = 310.15 K, $c_{Fe^{3+}}$ = 2.5·10^{-4} mol L^{-1}, c_{ligand} = 2.4·10^{-4} mol L^{-1}.

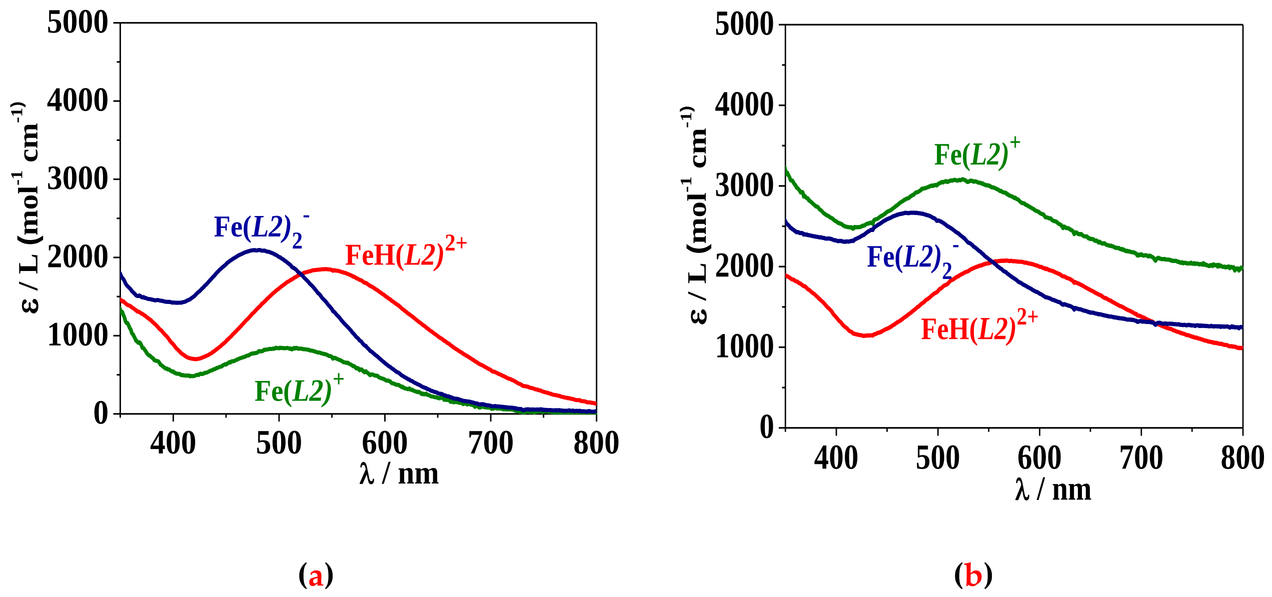

Figure S5. Graphical representation of calculated molar absorptivity of Fe^{3+}/H_2(*L2*) species at *T* = 298.15 K (**a**) and 310.15 K (**b**), *I* = 0.15 mol L^{-1} in $NaCl_{(aq)}$.

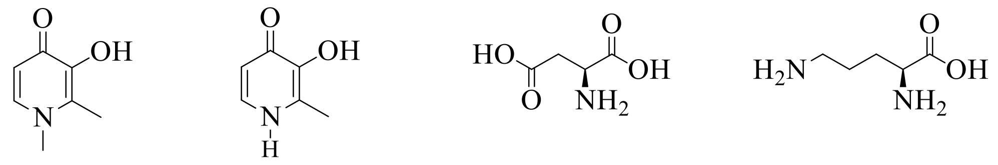

DFP H_2(*S1*) *Asp* *Orn*

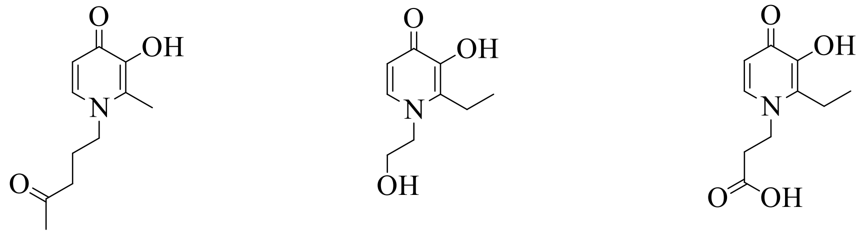

AcNPrHP H_2(*S2*) H_2(*S3*)

Figure S6. Molecular structures of compounds with similar structures and functional groups with respect to 3-hydroxy-4-pyridinones.

Abbreviations: *DFP* = Deferiprone; *Asp* = *L*-Aspartic acid; *Orn* = *L*-Ornithine; *AcNPrHP* = 1-(3′-methylcarboxyaminopropyl)-3-hydroxy-2-methyl-4-pyridinone; H_2(*S1*) = 3-hydroxy-2-methylpyridin-4(1H)-one; H_2(*S2*) = 3-hydroxy-1-(2-hydroxyethyl)-2-methylpyridin-4(1H)-one; H_2(*S3*) = 3-(3-hydroxy-2-methyl-4-oxopyridin-1(4H)-yl)propanoic acid.

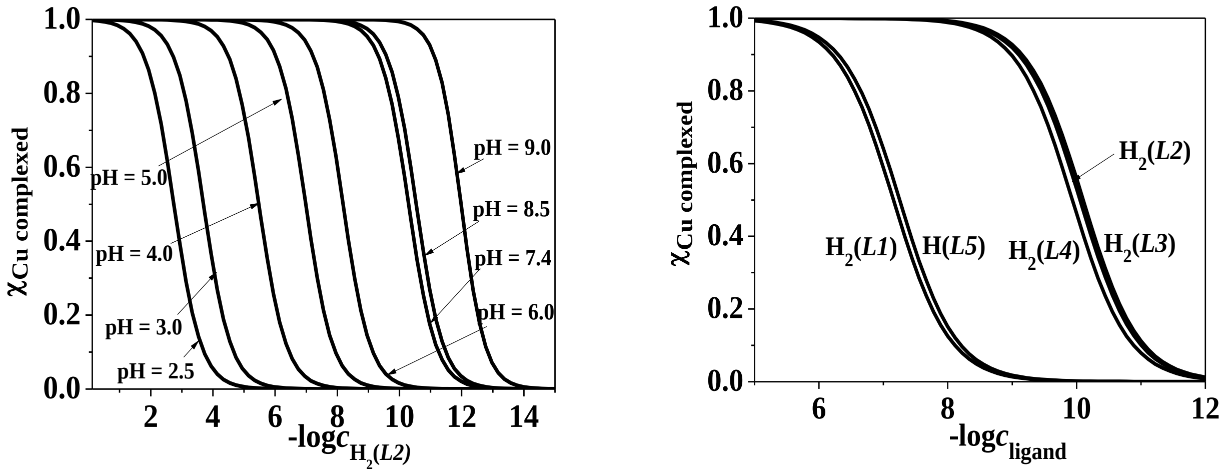

(a) **(b)**

Figure S7. Sequestration diagrams of: **(a)** Cu^{2+}/H_2(*L2*) species at *I* = 0.15 mol L^{-1} in $NaCl_{(aq)}$ and *T* = 298.15 K and different pHs, p$L_{0.5}$ values: 2.74 (pH = 2.5), 3.70 (pH = 3.0), 5.48 (pH = 4.0), 6.99 (pH = 5.0), 8.18 (pH = 6.0), 10.29 (pH = 7.4), 10.54 (pH = 8.1), 11.98 (pH = 9.0); **(b)** Cu^{2+}/(3,4-HPs) systems at the same ionic strengths and temperature, pH = 7.4. p$L_{0.5}$ values: 7.09 (H_2(*L1*)), 10.29 (H_2(*L2*)), 10.30 (H_2(*L3*)), 9.90 (H_2(*L4*)), 7.25 (H(*L5*)).

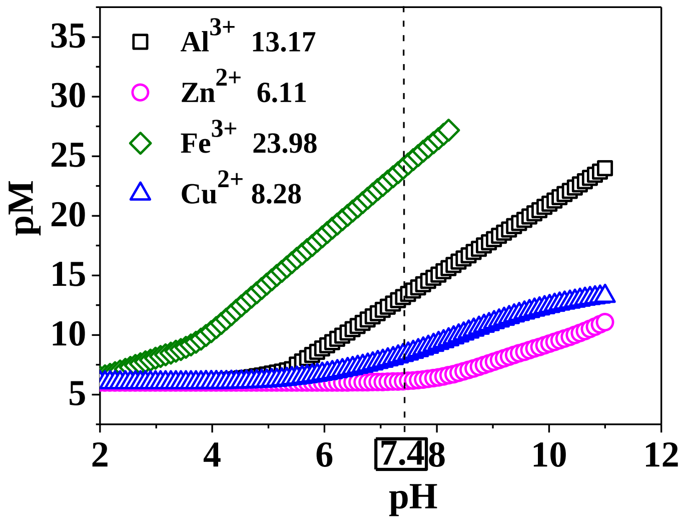

Figure S8. Calculated pM values *vs.* pH for the different M^{n+}/H(*L5*) systems at T = 298.15 K, I = 0.15 mol L^{-1} in $NaCl_{(aq)}$, $c_{M^{n+}}$ = 1.0·10^{-6} mol L^{-1} and c_{ligand} = 1.0·10^{-5} mol L^{-1}.
